# Dictionary learning allows model-free pseudotime estimation of transcriptomic data

**DOI:** 10.1186/s12864-021-08276-9

**Published:** 2022-01-15

**Authors:** Mona Rams, Tim O.F. Conrad

**Affiliations:** 1 Freie Universitaet Berlin, Arnimallee 6, Berlin, 14195 Germany; 2 Konrad-Zuse-Zentrum für Informationstechnik Berlin, Takustraße 7, Berlin, 14195 Germany

**Keywords:** Pseudotime estimation, Trajectory inference, Dimension reduction, Dictionary learning, Time course, Dynamic, Branching, Biomarker, RNA-seq, Single-cell

## Abstract

**Background:**

Pseudotime estimation from dynamic single-cell transcriptomic data enables characterisation and understanding of the underlying processes, for example developmental processes. Various pseudotime estimation methods have been proposed during the last years. Typically, these methods start with a dimension reduction step because the low-dimensional representation is usually easier to analyse. Approaches such as PCA, ICA or t-SNE belong to the most widely used methods for dimension reduction in pseudotime estimation methods. However, these methods usually make assumptions on the derived dimensions, which can result in important dataset properties being missed. In this paper, we suggest a new dictionary learning based approach, dynDLT, for dimension reduction and pseudotime estimation of dynamic transcriptomic data. Dictionary learning is a matrix factorisation approach that does not restrict the dependence of the derived dimensions. To evaluate the performance, we conduct a large simulation study and analyse 8 real-world datasets.

**Results:**

The simulation studies reveal that firstly, dynDLT preserves the simulated patterns in low-dimension and the pseudotimes can be derived from the low-dimensional representation. Secondly, the results show that dynDLT is suitable for the detection of genes exhibiting the simulated dynamic patterns, thereby facilitating the interpretation of the compressed representation and thus the dynamic processes. For the real-world data analysis, we select datasets with samples that are taken at different time points throughout an experiment. The pseudotimes found by dynDLT have high correlations with the experimental times. We compare the results to other approaches used in pseudotime estimation, or those that are method-wise closely connected to dictionary learning: ICA, NMF, PCA, t-SNE, and UMAP. DynDLT has the best overall performance for the simulated and real-world datasets.

**Conclusions:**

We introduce dynDLT, a method that is suitable for pseudotime estimation. Its main advantages are: (1) It presents a model-free approach, meaning that it does not restrict the dependence of the derived dimensions; (2) Genes that are relevant in the detected dynamic processes can be identified from the dictionary matrix; (3) By a restriction of the dictionary entries to positive values, the dictionary atoms are highly interpretable.

**Supplementary Information:**

The online version contains supplementary material available at (10.1186/s12864-021-08276-9).

## Background

The transcriptomic profile of a cell changes over time, for example during its development or when exposed to some (external) condition. Gene expression profiling can help to understand the underlying mechanisms, identify key genes in these processes, distinguish and characterise variants of different subgroups and more.

First approaches studying this time dynamic behaviour are ordering bulk samples based on expression similarity [1–3]. In this context, the term *pseudotime* was introduced, which describes a cells’ biological progression. In current pseudotime estimation experiments, single-cell datasets – in contrast to bulk datasets – are commonly analysed. The data of each single-cell is interpreted as a snapshot of the temporal development.

Since the development of single-cell RNA-sequencing (scRNA-seq), various pseudotime estimation methods have been published. An extensive review on 45 existing pseudotime estimation methods together with an evaluation on a total of 339 datasets is given by Saelens et al. in [4]. Typically, these methods start with a dimension reduction step to allow for easier handling of the data. Three methods that belong to the most widely applied approaches for dimension reduction in pseudotime estimation are: (1) Principal component analysis (PCA) [5], (2) independent component analysis (ICA) [6], and (3) t-distributed stochastic neighbour embedding (t-SNE) [7]. Well known pseudotime estimation methods using PCA for dimension reduction are SCOUP [8], TSCAN [9], and Waterfall [10]. Monocle [11] presents a well-known method using ICA and SCUBA [12] uses t-SNE.

Both, for ICA and PCA, the derived components – which the data is projected onto – have to satisfy certain assumptions, e.g. independence (in ICA) or orthogonality (in PCA). Data compression using PCA premises that the desired information is exactly provided by variance. However, other factors, caused for example by sampling or technical bias, can have a large impact on the variance of the data as well. Further – in the light of the application of this paper – some genes might vary little, but are important for the investigated process - or vice versa, the biological question might not be related to the highest variance in the data. ICA presents an alternative approach to PCA. It derives statistically independent components, which is a stronger condition than non-correlation in PCA. It is the nature of biological processes, however, that they are connected and depend on each other. Also, processes relevant for dynamic changes might indeed be dependent. Consequently, these processes could be missed by ICA and pseudotime estimation based on a low-dimensional representation from ICA would be misleading.

The third mentioned approach for dimension reduction of transcriptomic data is t-SNE. This is a non-linear approach and thus does not result in two matrices, like PCA, ICA, and other matrix factorisation approaches. This means, that an interpretation of the result is more difficult, and it is more difficult or impossible to derive the genes that are governing the dynamics. Further, t-SNE focuses on preserving local structures, which can be misleading when pseudotimes of the entire dataset are requested because global distances are relevant in this case.

Concerns regarding the use of these methods for the analysis of transcriptomic data have been mentioned in [13–15]. Given the variety of available approaches for dimension reduction, Street et al. [16] present their cell lineage and pseudotime inference method Slingshot, which allows performing the dimension reduction step with any method suitable for this purpose. Together, this indicates (the need for) a rethinking, away from the use of the same old default approaches for dimension reduction in pseudotime estimation and the need for the development of a dimension reduction approach which projects data to biologically meaningful components.

In this paper, we apply dictionary learning (DiL) for dimension reduction of transcriptomic datasets from dynamic processes. In DiL, the data matrix is factorised into a product of two matrices, a dictionary and a matrix of coefficient vectors for each sample. The dictionary columns, so-called atoms, do not need to satisfy any assumptions among each other, which presents a major difference to PCA and ICA. Therefore, we say that our approach is model-free. Compared to representations derived from PCA or ICA, those from DiL are potentially better because the vectors are on average nearer to the signal examples due to the refrain from the assumptions [17].

The representation derived with DiL is highly interpretable: Each atom contains values that can be assigned to each gene. These atoms can therefore be interpreted as gene modules. Gene modules are groups of genes that relate to proteins that interact to coordinate specific cellular functions and biochemical events. Genes corresponding to high entries in an atom can be interpreted as such a module. In our previous study [18] we have shown that the gene modules derived from a DiL analysis of transcriptomic datasets are biologically relevant. The coefficient vectors assign weights to those modules for each sample, resulting in a low-dimensional representation. DiL is designed to yield sparse representations. This way, atoms are constructed such that each sample requires as few atoms as possible to be reconstructed. When applying DiL to transcriptomic data, our idea is that this leads to atoms, respectively modules, that are relevant to processes in many samples.

In our previous study [18] we use a DiL based approach for transcriptomic data compression and gene module detection, further referred to as DiL For The Analysis Of Transcriptomic Datasets (DLT). We show that, applied to data from different types, (1) DLT yields gene modules that agree with the biological context of the distinctive types, while (2) type-specific differences are maintained in the sparse low-dimensional representations.

Using a modification of DLT for the analysis of dynamic transcriptomic datasets, further referred to as dynDLT, we aim to find modules that represent gene modules that are relevant in the detected dynamic processes. An analysis of these gene modules can provide deeper insight into the cellular dynamic processes. The coefficient vectors entail the coefficients corresponding to these modules. This way, we use the coefficients to interpret them as the pseudotemporal ordering.

In this paper, we first perform a simulation study. We analyse simulated datasets in which a subset of genes expresses a characteristic pattern over simulated time. We evaluate two things: (1) Whether the coefficient vectors preserve the simulated patterns in low-dimension and (2) the overlap of genes in the modules with the genes exhibiting the simulated patterns. dynDLT accurately represents the simulation patterns and genes. Results from dynDLT are compared to results from ICA, non-negative matrix factorisation (NMF), PCA, t-SNE, and Uniform Manifold Approximation and Projection (UMAP). Compared to the other methods, dynDLT reaches the highest performance for the majority of the evaluated datasets. ICA and NMF are the two approaches with the most similar performance.

In addition to the simulation study, we analyse 8 real-world time course datasets. Among these, 2 datasets contain samples from one phenotype and 6 datasets are composed of samples from different subtypes. Subtypes can for example be similar cells that are exposed to different conditions or stem cells that develop into specific cell types. Such datasets are often referred to as having branching timelines. We evaluate whether they are represented by the coefficients from our dynDLT. While in our previous study [18] we showed that DLT represents data from different sample types well, in this paper, we thus extend DLT by adding time dynamics. To enable an evaluation of the results in a meaningful way, we analyse datasets for which the experimental times are known. This allows for a comparison of the experimental times with the estimated pseudotimes.

For the real-world data analysis, the evaluated methods perform similarly well. Nevertheless, dynDLT is overall performing slightly better. However, for these datasets large groups of samples have the same timestamp, meaning that time labels are not known in detail. Therefore, we assign more importance to the experiments on simulated datasets, as they allow for a better evaluation of the methods’ performance.

## Results

To gain a deep understanding of our method dynDLT for pseudotime estimation of transcriptomic data, a simulation study is performed as a first analysis. Subsequently, 8 real-world dynamic transcriptomic datasets are analysed with dynDLT. All results are compared to those from independent component analysis (ICA), non-negative matrix factorisation (NMF), principal component analysis (PCA), t-distributed stochastic neighbour embedding (t-SNE), and Uniform Manifold Approximation and Projection (UMAP).

For the simulated and real-world data we present results for dynDLT, in which the pseudotimes are derived directly based on the coefficient vectors returned from dynDLT, respectively the comparison methods. In addition, for the real-world data we also present results when the dimension reduction step in Monocle is performed with dynDLT. The pseudotimes are then estimated from the resulting manifold. Note that Monocle is chosen exemplary, and any other method might be chosen as well.

### Simulation studies

In this section, results from dynDLT for multiple simulated datasets are evaluated. In each simulated dataset, a subset of the genes exhibits a characteristic pattern over simulated time (details on the simulated datasets are given in “Simulated data” section). To assess the performance of the dynDLT results for the simulated datasets, two things are evaluated: (1) Whether the coefficient vectors preserve the simulated patterns in low-dimension and (2) the overlap of marker genes derived from the dictionary atoms with the genes exhibiting the simulated patterns (marker genes are defined in “Estimating pseudotime based on dictionary learning” section).

**Fig. 1:**
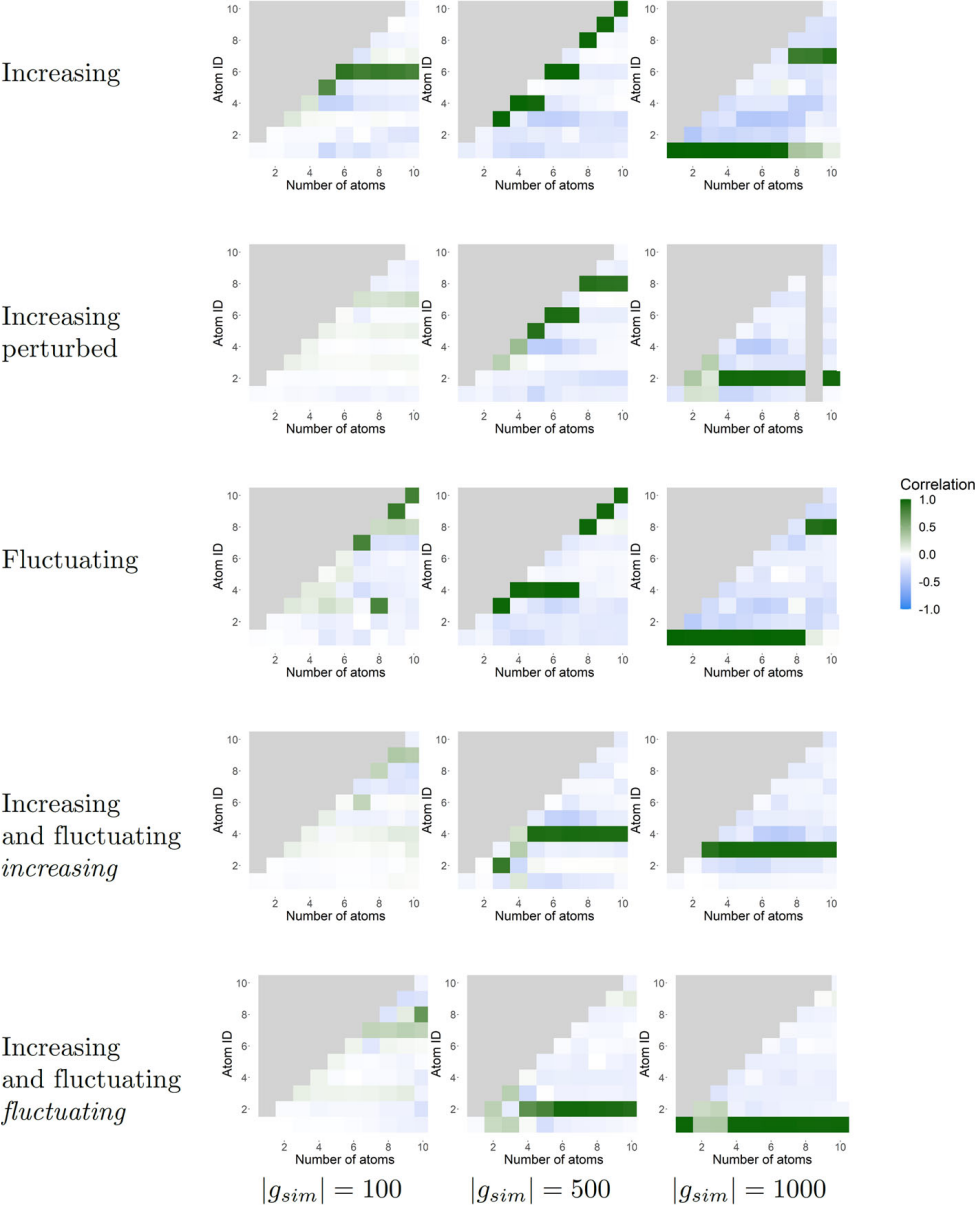
Spearman correlation for the values of the coefficient vector from DiL for each parameter. Shown are results for five simulation (sub-)pattern/ perturbation combinations in rows for three values of simulated genes exhibiting the pattern (|*g*_*sim*_|) in columns. The dataset labelled ‘Increasing perturbed’ is the one with *high-noise*-and-*zero-counts* perturbation, for the other datasets only *noise* perturbation is conducted. For the dataset with an increasing and a fluctuating pattern, the correlations for each subpattern are shown separately. The respective subpattern is given in the row description in italic letters. The x-axis of each plot shows the number of atoms (*m*) and the y-axis the atom ID. The maximal correlation increases or remains stable for an increase of the presented values for |*g*_*sim*_| (100,500,1000) with few exceptions. Typically, for each value of *m* there is one atom that represents the time course

To evaluate the coefficient matrices, the Spearman correlation of the coefficients for each atom with the simulated patterns are investigated. The influence of the parameter *m*, the number of dictionary atoms, which is varied over *m*∈[1,...,10] is considered first. Correlations close to 1 are reached for the majority of datasets when *m* is sufficiently large, e.g. *m*>3 (see Fig. 1). Note, that this also depends on the number of genes exhibiting the simulated pattern, |*g*_*sim*_|, and the intensity of perturbation, which is discussed in the next paragraph. For an increasing number of atoms, once a high correlation is reached, it remains high for larger values of *m*. Generally, for each dictionary, there is one atom that represents the simulated pattern.

**Fig. 2:**
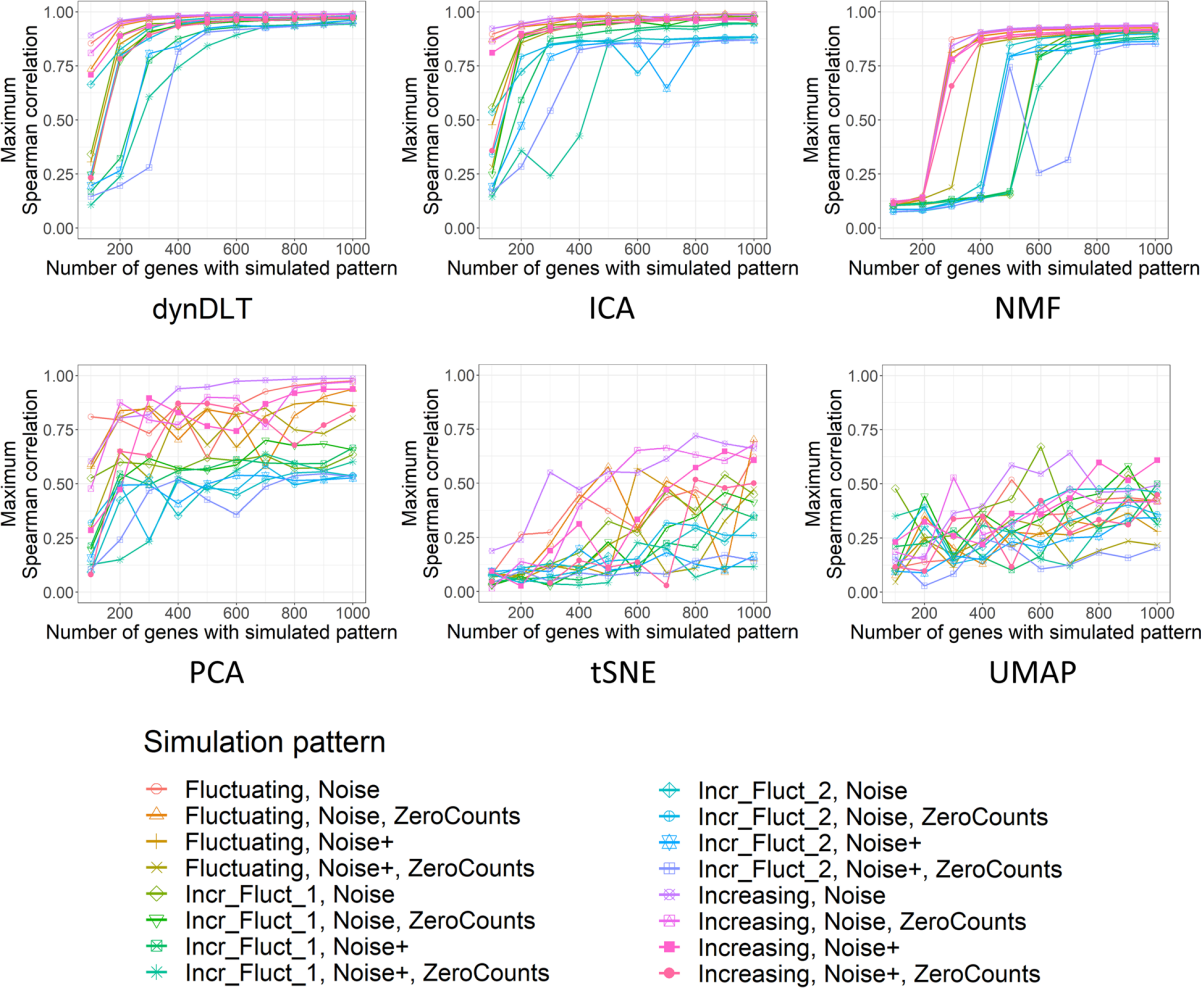
Correlation evaluations for the simulated datasets for each evaluated method. The subfigures shows evaluations for each method (method name in subtitle). The x-axis shows the number of genes with the simulated time pattern, |*g*_*sim*_|. The y-axis shows for each dataset the maximum correlations among the matrices with different method parameters. The dataset with one half of |*g*_*sim*_| increasingly ordered and the other half fluctuating is labelled “Incr_Fluct”, with “Incr_Fluct_1” being the half of increasing values and “Incr_Fluct_2” the half of fluctuating values. The *high noise* perturbation is labelled *Noise+*. Correlations for DiL, ICA, and NMF increase or remain similar for increasing |*g*_*sim*_|, which presents an anticipated behaviour. However, compared to DiL and ICA, correlations for NMF are high for higher values of |*g*_*sim*_| only. For PCA, t-SNE, and UMAP this property is generally not held

The atom with the maximum Spearman correlation to the ground truth is further considered for any value of *m*∈[1,...,10]. Correlations for dynDLT increase or remain stable for increasing |*g*_*sim*_|, the number of genes exhibiting the simulated patterns, (see Fig. 2), which presents an anticipated property, as the patterns are more significant when they appear in a larger number of genes. For |*g*_*sim*_|≥400 correlations are larger than 0.74 for all datasets. For the datasets with one half of genes increasing and the other half fluctuating over time, correlations are lower. Since for these datasets only |*g*_*sim*_|/2 genes exhibit each subpattern, the results can be compared to the other datasets with |*g*_*sim*_|/2 genes exhibiting the simulated pattern. Correlations are then similar. Hence, dynDLT does also identify two subpatterns well. Further, regarding the perturbations, *noise* and *noise-and-zero-counts* have a smaller effect than *high noise*. The *high-noise-and-zero-counts* perturbation leads to only a slightly decreased performance compared to *high noise*. We conclude from this that *zero-counts* are not problematic for dynDLT.

**Fig. 3:**
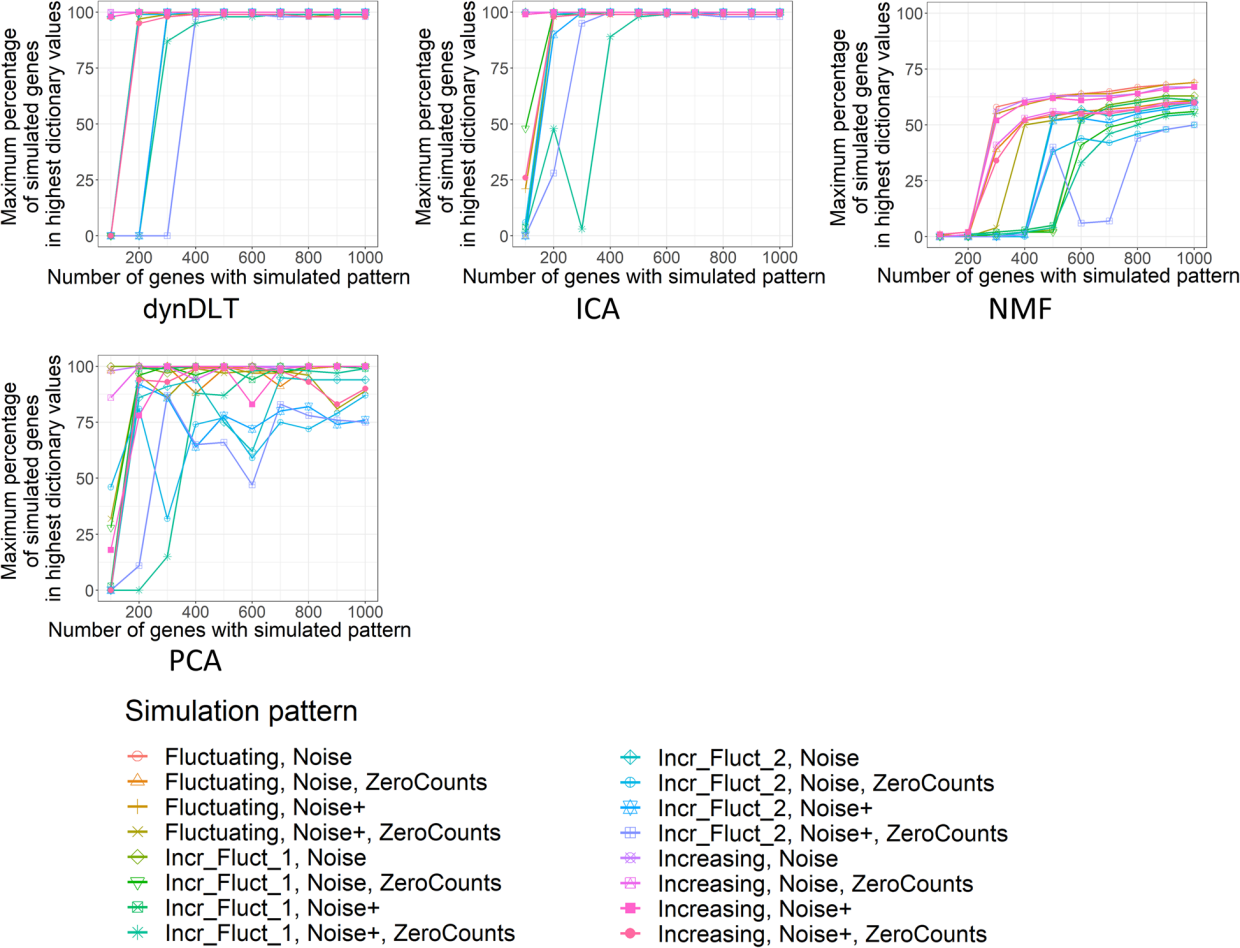
Percentage of correctly identified genes for the simulated datasets. The subfigures show evaluations for each method (method name in subtitle). The x-axis shows the number of genes with the simulated time pattern, |*g*_*sim*_|; The y-axis shows for each dataset the maximum percentage among the matrices with different method parameters. The dataset with one half of |*g*_*sim*_| increasingly ordered and the other half fluctuating is labelled “Incr_Fluct”, with “Incr_Fluct_1” being the half of increasing values and “Incr_Fluct_2” the half of fluctuating values. The *high noise* perturbation is labelled *Noise+*. DiL and ICA perform similarly well, whereas NMF does not reach as high percentages as the other methods. For PCA, high percentages are reached for most datasets, but they are mostly smaller than for DiL or ICA. Notably, for several datasets PCA-percentages decrease for increasing |*g*_*sim*_|, which presents an undesired behaviour

Regarding the use of dynDLT as a method for marker gene detection, we evaluate the percentage of the |*g*_*sim*_| highest dictionary entries overlapping with the *g*_*sim*_ genes (see Fig. 3). For all datasets for which |*g*_*sim*_|>300 the overlap is larger than 98% (median percentage for these datasets is 100, mean 99.6). This presents a very good performance and means that dynDLT is suitable for the identification of marker genes in the simulated dynamic processes. Those datasets that have a smaller overlap for |*g*_*sim*_|≤300 are either those with two subpatterns or those with *high noise* perturbation. This finding coincides with the conclusions from the correlation analysis.

#### Comparison methods

The results from dynDLT are compared to results from independent component analysis (ICA), non-negative matrix factorisation (NMF), principal component analysis (PCA), t-distributed stochastic neighbour embedding (t-SNE), and Uniform Manifold Approximation and Projection (UMAP). For simplicity, we subsequently refer to each column/ component/ dimension of the resulting dictionary-like matrices as component for all comparison methods. To evaluate the coefficient matrices, the Spearman correlation of the coefficients for each component with the simulated patterns are investigated. To evaluate the performance of each method for marker gene detection, the percentage of genes from *g*_*sim*_, the genes exhibiting the simulated pattern, in the |*g*_*sim*_| highest dictionary-like matrix entries per component is computed (see “Performance evaluation” section). Same as for dynDLT, for each dataset and method we present results for the best performing component among all components among all evaluated dimensionalities (for dynDLT this is the highest value among all values presented in each subfigure of Fig. 1, likewise for ICA in Additional file 1, Figure S1). Results are shown in Figs. 2 and 3.

Correlations with the simulated patterns for ICA and NMF increase or remain stable for increasing |*g*_*sim*_| with few exceptions (see Fig. 2), which presents an anticipated property that also appears in the dynDLT results. However, correlations for NMF are high for higher values of |*g*_*sim*_| only, especially for the dataset with two subpatterns, whereas dynDLT and ICA reach correlations close to 1 for small values of |*g*_*sim*_| and remain high. Correlations for PCA, t-SNE, and UMAP are to a large extent smaller than those of the other methods and, contrary to anticipation, do not increase when |*g*_*sim*_| is increased.

Taking a closer look at the difference of correlations with the simulated patterns between ICA and dynDLT, unlike dynDLT, for ICA, not all dimensionalities larger than the dimensionality that does first reach a high correlation yield a high correlation too (compare Fig. 1 and Additional file 1, Figure S1). This means, that for ICA multiple parameters have to be evaluated, and the best solution has to be selected. However, this requires a measure to select the best solution. Recall, that for dynDLT for increasing dimensionality once a high correlation is reached for one atom, this maintains stable for higher dimensionalities (compare Fig. 1 and Figure S1 in Additional file 1). In dynDLT, we therefore only need to select the one best performing atom, which should not be difficult as we see that one atom is representing the pattern clearly. However, for ICA we need to evaluate multiple dimensionalities and find those which perform best. Likewise as for dynDLT, among these, the best performing component has to be identified. If only a few data labels are known, this task becomes non-trivial, if not impossible. Nevertheless, we further assume one knows how to select the best parameter for ICA.

Correlation results are shown in Fig. 2. DynDLT reaches a higher performance than ICA in 64% of the evaluations, for 4% dynDLT and ICA perform identical and for 31% ICA performs better than dynDLT. On average, dynDLT outperforms ICA in the datasets with two subpatterns (average correlation difference 0.03) as well as those with perturbations (average correlation difference 0.01). ICA is slightly better in the datasets with |*g*_*sim*_|≤200 (average correlation difference 0.05). However, for datasets with |*g*_*sim*_|≥300 dynDLT is on average outperforming ICA also in these datasets (average correlation difference 0.03).

The performance of the methods when evaluating the percentages of correctly identified genes are similar to the performances for the correlation evaluations: DynDLT and ICA perform best and percentages increase or remain similar for increasing |*g*_*sim*_| with few exceptions. This is not the case for PCA. However, a major difference in the evaluation of the percentages of correctly identified genes and the correlation evaluations appears for NMF. NMF performs significantly worse than dynDLT and ICA when considering the percentages : Maximal percentages for NMF are 69, whereas the other three methods reach 100 for many datasets.

Comparing the percentages of correctly identified genes between the two best-performing methods ICA and dynDLT, both methods perform similar, especially when |*g*_*sim*_|>300 for which the maximal difference between the percentages is 6, with an average difference of 0.02. For |*g*_*sim*_|=300, dynDLT is performing better for the increasing pattern half of the dataset with two subpatterns over time and *high noise* as well as *zero-counts* perturbation. ICA, on the other hand, performs better for the fluctuating pattern half of this dataset.

#### Conclusions from the simulation study

In the simulation study 160 simulated (sub-)pattern/perturbation/ |*g*_*sim*_| combinations are analysed in total (note, that each subpattern of the dataset with two subpatterns is considered separately, therefore we have 160 and not 120 in total). We start by summarising the influence of the parameter |*g*_*sim*_|, the number of genes exhibiting the simulated pattern: For its increase, meaning that the pattern becomes stronger in the dataset, we expected to see an increase or stability in the performance. Among the evaluated methods, only dynDLT, ICA, and NMF show this property with few exceptions for some values of |*g*_*sim*_|.

Overall, dynDLT detects simulated patterns well for either of the simulated patterns and also with perturbation. Generally, one of the dictionary atoms reflects the simulated pattern. The correlations of the coefficient vectors with the simulated patterns are > 0.9 in 120 of 160 evaluations. The average correlation over all evaluations is 0.87 (median 0.96). The percentage of marker genes overlapping with the simulated genes is > 90*%* in 144 of 160 evaluations. Among the 112 datasets for which the |*g*_*sim*_|>300, for 106 datasets the correlation is > 0.9 and the percentage is > 90*%*.

ICA presents the only method that performs similarly good as dynDLT. However, dynDLT scores better for the majority of datasets. A higher performance of dynDLT is reached especially in datasets with two subpatterns over a wide range of simulation parameters, while ICA scores better for some datasets for which simulation parameter |*g*_*sim*_|<300. Further, regarding the performance over an increase of the dimensionality, unlike dynDLT, ICA results do not maintain good performance for all higher dimensionalities once the good performance is reached. Thus, for ICA multiple parameters have to be evaluated and a measure to select among those is required, which is non-trivial.

While NMF reaches high correlations similar to ICA and dynDLT – however, only for large values of |*g*_*sim*_| – it does not reach as high percentages as dynDLT and ICA for any value of |*g*_*sim*_|. Results for t-SNE and UMAP are significantly worse.

### Pseudotime estimation for dynamic real-world data

The simulation studies in which expression time patterns are simulated reveal that the coefficient vectors from dynDLT reflect such patterns well. In this section, real-world time course datasets are analysed with dynDLT, and it is examined whether the dynamic changes are represented by the coefficient vectors. To evaluate the time representation, data from samples in dynamic processes taken at different time points is analysed. The time points are used for the evaluation of the estimated pseudotimes.

We start the real-world data analysis with an evaluation of 2 time dynamic datasets with samples from one phenotype/ experimental condition. Due to our findings in [18], which revealed that the compressed representations from DLT, our DiL based approach for the analysis of static transcriptomic datasets, maintain differences among samples from different phenotypes, we are also interested in such datasets. Therefore, we also analyse 6 dynamic datasets with samples from different subtypes. We compare the results from dynDLT to those from ICA, NMF, PCA, t-SNE, and UMAP (for details on the comparison approaches see “Comparison methods” section). For simplification, we refer to each column/ component/ dimension of the resulting dictionary(-like) matrices as component for all methods.

As described in “Estimating pseudotime based on dictionary learning” section - with examples in “Simulation studies” section - pseudotimes are derived based on one component of the low-dimensional representation for each method. Thereto, the order of the coefficients of a component for each sample is used as the pseudotemporal ordering. For each component of the resulting low-dimensional representation, the Spearman correlation with the experimental times is computed. Note, that the Spearman correlation measures univariate behaviour. In consequence, whenever a set of components captures the time dynamics, this does not become visible by the Spearman correlation. The component for which the highest correlation is measured is used for pseudotime estimation.

As an alternative approach, for the real-world data analysis, we combine the low-dimensional representations of dynDLT and the comparison methods with the polygonal reconstruction algorithm from Monocle. To derive a pseudotemporal ordering from the entire low-dimensional representation, this algorithm constructs a minimal spanning tree (MST) from the representation. Next, it determines the longest connected path within the MST. Last, each node (sample) is assigned to the closest point on this longest path. The pseudotimes are then derived by this path. This way, the entire low-dimensional representation is used for the pseudotime estimation and the selection of a component for the pseudotime estimation is no longer required.

Note that by comparing the derived pseudotimes to experimental time points, we treat the experimental times like the ground truth. However, this is not necessarily correct for each sample, particularly because multiple samples have the same time label. This is a crucial remark. Consequently, we do not expect to obtain correlations of 1. Nevertheless, the experimental times should provide an orientation to evaluate whether the time dynamics are captured in principle.

#### Results for time dynamic data from one type

**Fig. 4:**
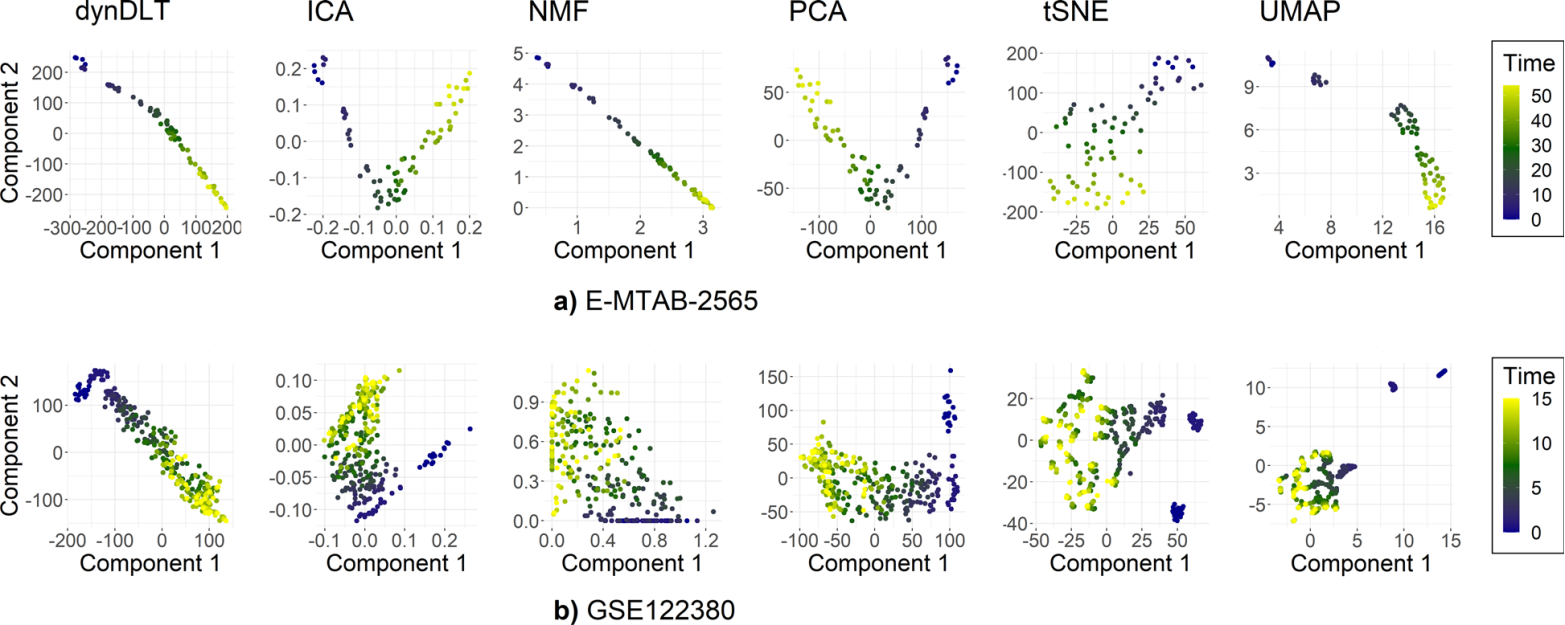
2D visualisation of the coefficients for all evaluated methods for two components for two datasets. Shown are visualisations of the coefficients for the two datasets without subtypes (dataset E-MTAB-2565 in Subfigure a and dataset GSE112004 in Subfigure b). The respective dataset ID is given in the subtitle. The data points are coloured based on the experimental time points (colour encoded in legend). The parameters for t-SNE, respectively UMAP (*perplexity*=10,*number*_*of*_*neighbours*=10) are chosen to maximise the correlation among all values evaluated on average for both datasets. As the varied parameter for the other methods is the dimensionality, which is set to 2 here, for these methods no parameter search is conducted. Hence, for the show representations a parameter study is performed for t-SNE and UMAP only. For both datasets, for many methods, at least one component is representing well the dynamics of the data. It is striking that the low-dimensional representations from dynDLT represent the dynamics with low noise

**Fig. 5:**
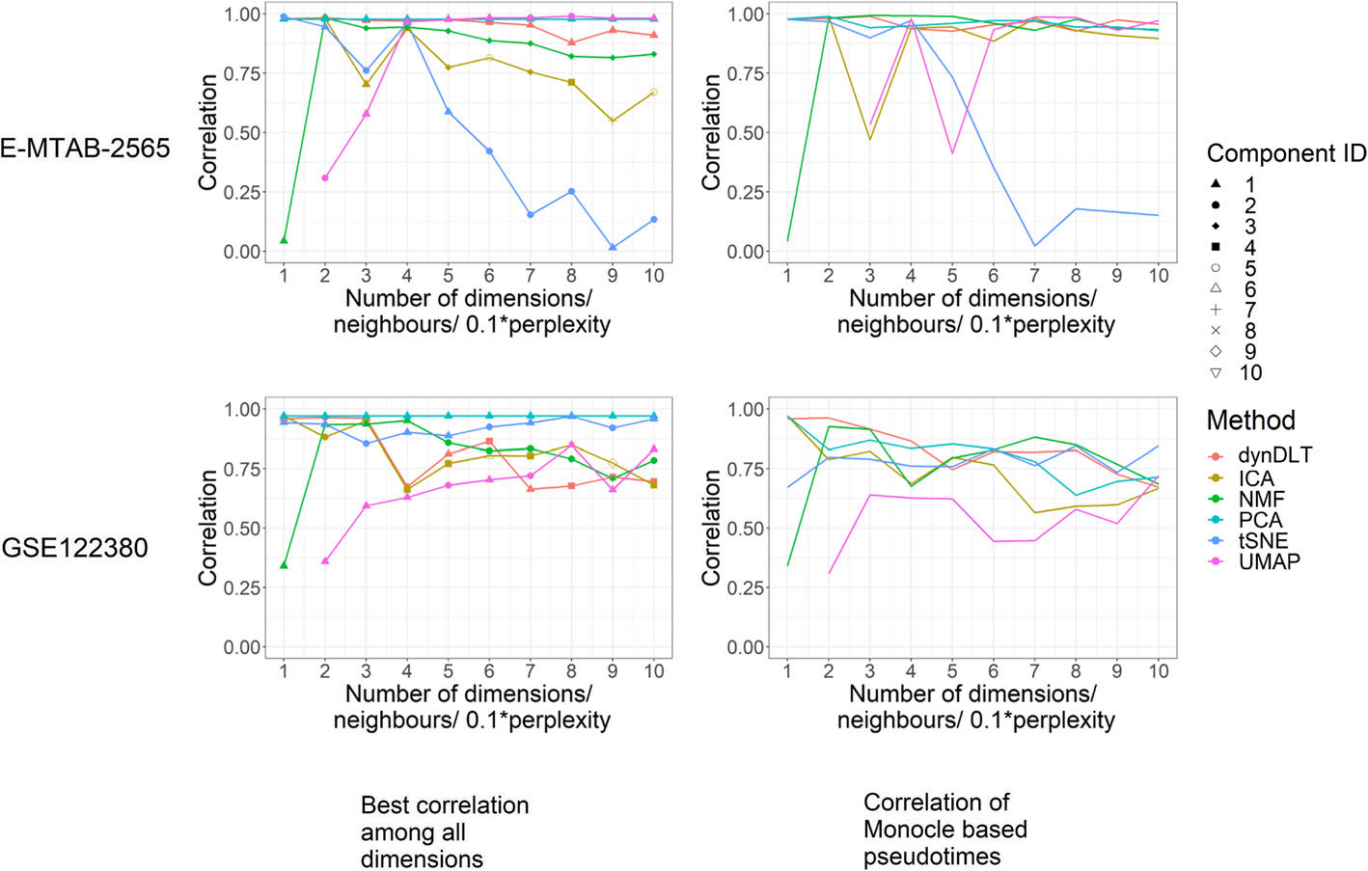
Correlations of experimental times with pseudotimes for 2 real datasets with samples from one phenotype. Subfigures to the left show results for pseudotimes derived based on the ranks of the values of the coefficient vector for the best scoring component; Subfigures to the right show the correlations for pseudotimes derived based on the entire low-dimensional representation with Monocle’s polygonal reconstruction. The x-axis displays the number of components the data is reduced to, or for UMAP the number of neighbours and for t-SNE the perplexity. The majority of correlations are higher when pseudotimes are derived based on one atom

For the 2 real-world datasets without subtypes, results for all methods with 2 components are visualised in Fig. 4. For these datasets, the correlations for the dynDLT results are maximal for dictionaries with few atoms. e.g. *m*≤3 (see Fig. 5). For dataset E-MTAB-2565 the highest correlation for dynDLT over all evaluated values for the number of atoms is 0.98, the smallest correlation is 0.80. PCA and ICA reach similar high correlations. For dataset GSE122380 the highest correlation for dynDLT is 0.88, the smallest correlation is 0.49. ICA, PCA, NMF, and UMAP reach similar high correlations.

As an alternative to a selection of the best scoring atom, we also derive the pseudotemporal ordering from the entire low-dimensional representation using the polygonal reconstruction approach from Monocle. For both datasets the correlations are similarly high as from one single component (see Fig. 5). However, especially for dataset GSE122380, the correlation drops for higher dimensions for several methods.

We have also conducted an analysis of the marker genes derived from the respective component that is displaying the pseudotime, as explained in “Performance evaluation” section. Recall that we used a significance cut-off of 10^−3^. The full list of the respective GO-terms is given in the supplementary material, Additional file 2.

For all methods, the resulting GO-terms contain terms that are either associated with dynamic cell processes, or with the sample types, respectively experimental conditions. For both datasets, only dynDLT and NMF identify genes for which the GO-terms can be associated with the sample types, respectively experimental conditions. This is in line with the property of these two methods as being those with positive dictionary(-like) matrices, which enhances interpretability. Comparing between dynDLT and NMF, the proportion of terms regarded as meaningful is higher in dynDLT. For NMF, more terms are found, however, many of them are quite general.

#### Results for time dynamic data with different subtypes

**Fig. 6:**
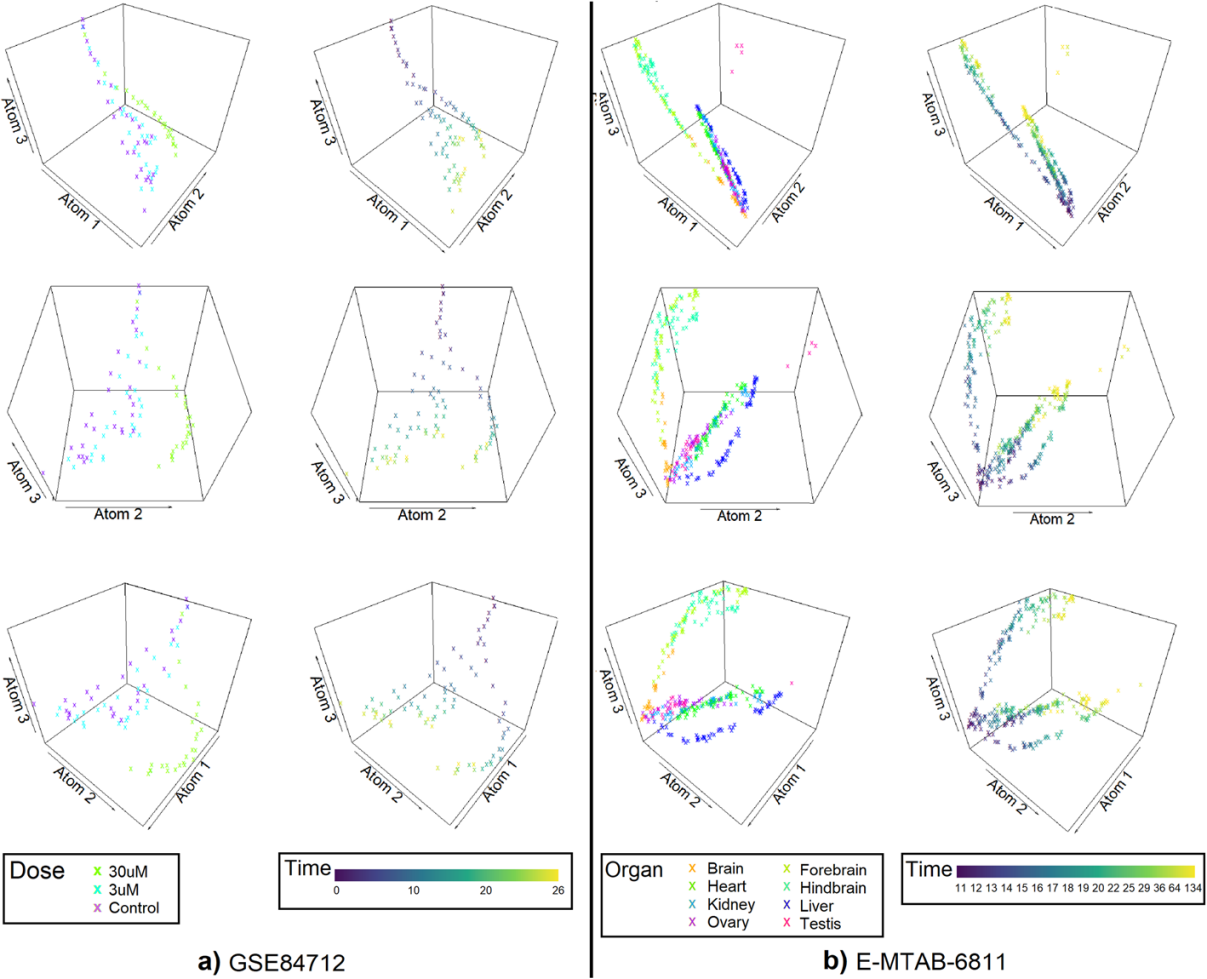
3D visualisation of the coefficients for a dictionary with three atoms for two datasets. Shown are three rotations of the 3D visualisation. Visualisation for dataset GSE84712 are shown on the left; Visualisations for dataset E-MTAB-6811 are shown on the right. For each dataset, the low-dimensional representation is coloured once by the subtype separating feature (Dose for GSE84712 and Organ for E-MTAB-6811) and once by the experimental times. For dataset GSE84712, samples with a dose of 30 *μ*M are clearly separated from those with no or 3 *μ*M lead exposure for higher times. On each branch, samples are well ordered according to experimental times. A similar pattern is observable for dataset E-MTAB-6811

The analysis of real-world data with time dynamics reveals that the coefficient vectors from dynDLT have a high correlation with the experimental times. In this section, results for 6 datasets with different subtypes are presented. The datasets are analysed with dynDLT (see Fig. 6) and the comparison approaches ICA, NMF, PCA, t-SNE, and UMAP.

The metadata of the 6 datasets with subtypes contains several features (compare Table 1 and Additional File 1, Table S1). Each of these features is regarded as a candidate variable for a subtype partition with different time patterns. To evaluate whether the low-dimensional representation displays the times of the feature-subtypes well, for each subtype, the correlation of the experimental times and values of the coefficient vector are computed for all samples belonging to the subtype. To merge all subtype values for each feature and come up with a value for the entire feature, each of these correlations is scaled by the percentage of samples that belong to the subtype. The resulting values are summed for each feature (for details see Additional File 1, section S2.3).

**Table 1:** Overview of the metadata for the 8 real-world datasets (after outlier removal)

Database-ID	Database	Datatype	Organism	Samples	Reads/Genes	Time points	Metadata variables
GSE122380	GEO	scRNA-seq	Homo sapiens	294	16,237	16	-
E-MTAB-2565	ArrayExpress	Microarray	Arabidopsis thaliana	71	20,361	18	-
GSE100425	GEO	RNA-seq	Mus musculus	120	19,715	7	6
GSE129486	GEO	RNA-seq	Homo sapiens	174	30,566	9	6
GSE84712	GEO	scRNA-seq	Homo sapiens	78	18,255	27	2
GSE87375	GEO	scRNA-seq	Mus musculus	912	22,027	7	8
GSE92652	GEO	RNA-seq	Homo sapiens	92	46,378	6	5
E-MTAB-6811	ArrayExpress	Microarray	Rattus norvegicus	359	27,330	16	4

The first two datasets are those without subtypes

**Fig. 7:**
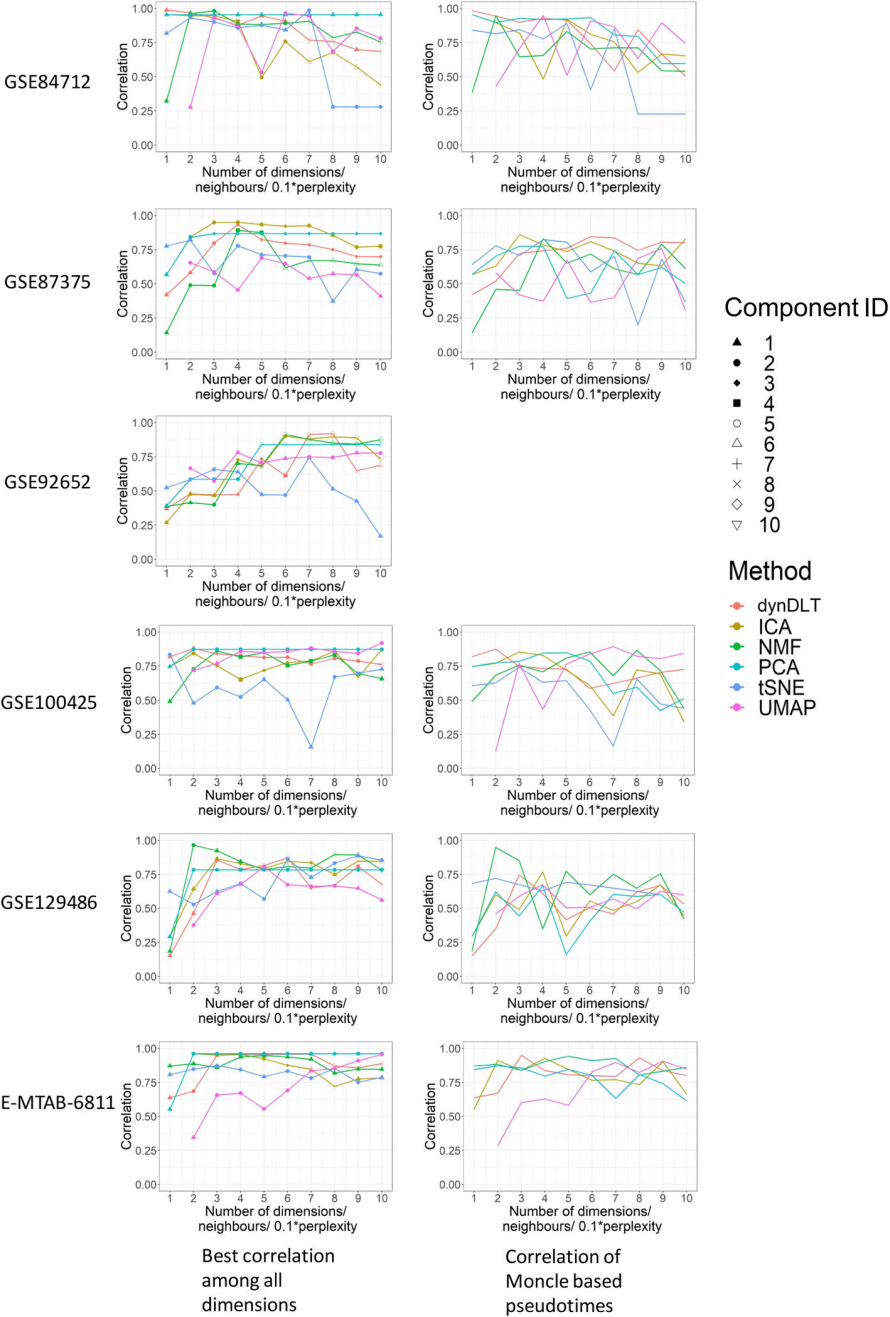
Correlations of experimental times with pseudotimes for 6 real-world datasets with multiple subtypes. Subfigures in each row show evaluations for each dataset. Subfigures to the left show results for pseudotimes derived based on the ranks of the values of the coefficient vector for the best scoring component; Subfigures to the right show the correlations for pseudotimes derived based on the entire low-dimensional representation with Monocle’s polygonal reconstruction. For dataset GSE92652 Monocle fails, which is why only the remaining 5 datasets are analysed here. The x-axis displays the number of components the data is reduced to, or for UMAP the number of neighbours and for t-SNE the perplexity. The majority of correlations are higher when pseudotimes are derived based on one atom

When the pseudotimes are derived based on the ordering of the coefficients for each component, several methods reach high correlations for several datasets (see Fig. 7). Considering the maximal correlation over all parameters per dataset and method, dynDLT reaches the highest correlation for 3 out of 6 datasets (GSE84712, GSE92652, EMTAB6811). ICA and UMAP are among the best scoring methods for 2 datasets each (ICA: GSE87375, EMTAB6811; UMAP: GSE100425, EMTAB6811).

For many datasets, the correlations are high for several parameter values (number of components/ number of neighbours for UMAP/ perplexity for t-SNE). Solely the smallest parameter value evaluated is often leading to significantly worse results. For all higher parameter values for all datasets but GSE92652, correlations do on average not change by more than 0.23.

When the pseudotimes are derived by an integration of the entire low-dimensional representation from each method with the polygonal reconstruction from Monocle, the correlations are slightly worse than those for the single best component for several dataset/method combinations (see Fig. 7). For dataset GSE92652 Monocle fails, which is why only the remaining 5 datasets are analysed here. In this case, dynDLT reaches the highest correlations for 2 out of the 5 datasets. ICA, NMF, and UMAP are among the best methods for 1 dataset each.

We have also conducted an analysis of the marker genes derived from the respective component that is displaying the pseudotime, as explained in section 2. Recall that we used a significance cut-off of 10^−3^. The full list of the respective GO-terms is given in the supplementary material, Additional file 2. In the following, we refer to those GO-terms which are associated to dynamic processes, or those which are in association with the sample types, respectively the experimental set-up, as the *relevant* ones. For the individual datasets, the results are as follows:

For dataset GSE100425, for all methods but ICA two of the significant GO-terms are *relevant*. For ICA, six of the significant GO-terms are relevant. The number of total significant GO-terms is higher for ICA and NMF, compared to dynDLT and PCA, though. This means that the number of non-relevant GO-terms is also higher for these methods.

For dataset GSE129486, for dynDLT, four significant GO-terms are relevant. For all other methods this amount is smaller and the number of non-relevant GO-terms is significantly higher for NMF and PCA.

For dataset GSE84712 the 18 GO-terms are relevant for dynDLT. With 7 relevant GO-terms NMF is the method with the second highest number of relevant GO-terms. The number of non-relevant GO-terms is significantly higher for NMF and PCA. For ICA six out of eight GO-terms are relevant.

For dataset GSE87375 PCA is the only method for which more than one GO-term is significant.

For dataset GSE92652 the only significant GO-term that can be associated to the sample types, respectively the experimental set-up is found by NMF. However, for ICA, NMF, and PCA a number of GO-terms associated to dynamic processes are found. For this datasest the dynDLT results do not contain any such GO-terms.

For dataset EMTAB6811, the number of relevant GO-terms varies from two to three in all results but those found by NMF for which 13 such terms are found. However, at the same time, similar to the results for the other datasets, for NMF the amount of non-relevant GO-terms is a lot higher than for the other methods.

In summary, this pseudotime evaluation suggests that the results by the dynDLT method contain more relevant information compared to the other methods for the real-world data evaluations. Recall, however, that the labels for the analysed real-world datasets – which are used for the evaluation – are less detailed than those for simulated data with only a few time stamps per dataset. We would therefore like to highlight again the results of the simulated data analysis. Knowing the actual time points of each cell could give better insight when comparing with the estimated pseudotimes and might show an even more significant benefit for dynDLT. In the GO-term evaluation, for two out of six datasets, namely for GSE129486 and GSE84712, dynDLT is performing better than the other methods based on our evaluation criteria described above. Among the other dataset no method shows an outstanding performance for datasets GSE100425 and E-MTAB-6811. For dataset GSE87375, PCA is the best performing method. For dataset GSE92652 all comparison methods perform similar. Further, regarding the methods other than dynDLT, it is striking in particular for NMF that the proportion of relevant GO-terms in the significant GO-terms is comparably small.

## Discussion

We have introduced and evaluated our new approach dynDLT for pseudotime estimation of transcriptomic datasets which are composed of samples from dynamic processes. We started with a simulation study in which we focused on two things: (1) Whether the coefficient matrices preserve the simulated patterns in low-dimension and (2) the overlap of genes corresponding to the high dictionary values with the genes exhibiting the simulated patterns – which, if successful, allows for the interpretation of the dynamic processes on the gene level.

We have performed an extensive simulation study as well as an analysis of 8 real-world datasets. The low-dimensional representations from dynDLT represent the simulated time patterns well. At the same time, the marker genes from the dictionary significantly overlap with the genes that exhibit the simulated pattern. Hence, with regard to the simulation studies, dynDLT represents a method that is capable of pseudotime estimation, and it can be applied to find modules that entail the gene sets which play a relevant role in the dynamic processes.

We compared our results to those from other approaches used in pseudotime estimation, or those that are method-wise closely connected to dynDLT: ICA, NMF, PCA, t-SNE, and UMAP. ICA and NMF perform well in pseudotime estimation for the simulated datasets. However, for NMF, this is only the case for datasets with many genes exhibiting the time pattern. ICA, on the contrary, performs worse than dynDLT in the majority of evaluated datasets and especially when the simulated data contains more than one time pattern. For marker gene detection, only dynDLT and ICA perform well overall. PCA yields good marker gene detection results for some datasets only. As discussed throughout the paper, another advantage of dynDLT is that the dictionary entries are positive, unlike for ICA and PCA. This allows for an easier interpretation of the low-dimensional representation.

For the real-world data evaluation, high correlations between the experimental time points and the values of the coefficient vector are measured for all methods. However, summarising over all evaluated datasets, dynDLT has the best overall performance in this correlation analysis. Due to the few known time points for the real-world datasets, the assessment based on correlation is vaguer than for the simulated datasets, where the pseudotime ordering of each sample is known. Therefore, we put more emphasis on the simulation results and provide the real-world data results as a confirmation that pseudotime estimation based on dynDLT is also working on real-world data.

For the real-world datasets the identified gene-sets are evaluated by a GO-term analysis. Note though, that, among the analysed methods, the only methods suitable for this purpose are the linear ones, namely dynDLT, ICA, NMF, and PCA. For four out of eight datasets dynDLT is performing better than the comparison methods. For two further datasets no method shows an outstanding performance; For one dataset PCA is the best performing method. Additionally, it is striking that, in the results for the comparison methods and in particular for NMF, the proportion of relevant GO-terms in the significant GO-terms is small compared to dynDLT.

Note, that even though we have analysed datasets from multiple phenotypes with different experimental conditions among samples, this work does not present a method to identify branches. Notably, analysing such datasets and evaluating the pseudotemporal ordering of each branch shows that time patterns are captured for such multi-type datasets. A visualisation of the coefficient vectors derived from dynDLT shows that dynDLT is also well suited for the visualisation of dynamic transcriptomic datasets. For branch detection using dynDLT, the compression methods in existing branch detection approaches, e.g. PCA, ICA, t-SNE, can be substituted with dynDLT. As an example, we exchange ICA in Monocle with dynDLT which yields promising results. Certainly, we have only evaluated one method for the incorporation of the entire representation. A study of the incorporation of dynDLT into other approaches remains for future research.

A challenge in the application of the presented dynDLT based approach and the comparison approaches, when not combined with other algorithms, is to identify the atom/component that captures the dynamics, or how to otherwise incorporate all atoms/components. This is still unsolved.

In the application of dynDLT for pseudotime estimation, only one parameter (number of atoms, *m*) has to be selected by the user. In the majority of real-world data analyses, a value of *m*=3 yields good results. If no experiments are carried out, we suggest using this value.

## Conclusion

In this paper, we present a new dictionary learning based method for pseudotime estimation of simulated and dynamic real-world data, dynDLT. We compare the performance of dynDLT to those of ICA, NMF, PCA, t-SNE, and UMAP.

The simulation study shows that dynDLT is suitable for the detection of dynamic patterns in transcriptomic data. In addition, for the simulated datasets, the genes that are identified by our method to be important are in large agreement with the ground truth. When comparing to similar methods such as ICA, we find that ICA performs worse for the majority of the considered datasets, especially for those with more than one expression pattern. Further, ICA performs well for some parameter values only, which requires a parameter search and a measure to select the results with the best performance. This, however, is non-trivial in pseudotime estimation as it presents an unsupervised task.

For the real-world data analysis, dynDLT is overall performing slightly better than the other methods, based on the evaluation using correlation of the estimated times with the experimental time points.

We derive pseudotime from one atom of the dictionary given by dynDLT, respectively from one component of the dictionary-like matrices from other approaches. The identification of this one atom presents a challenge. To provide a workflow without having to detect this one atom, we additionally incorporate the entire low-dimensional representation into Monocle, which also yields good results.

The main advantages of using dynDLT for pseudotime estimation of dynamic transcriptomic datasets are: (1) It presents a model-free approach, meaning that the data representation is not required to fulfil specific assumptions, such as orthogonality or independence of the dimensions; (2) Important marker genes can be identified from the dictionary matrix; (3) By a restriction of the dictionary entries to positive values, the dictionary atoms are highly interpretable; (4) A good performance is reached for several parameter values (number of atoms), which means a large parameter search is not necessary.

## Methods

We apply Dictionary learning (DiL) for pseudotime estimation of transcriptomic datasets. DiL is a matrix factorisation approach that results in a dictionary matrix and a matrix carrying the coefficient vectors. In our previous study [18] we show that DiL can be applied to transcriptomic data when a small modification is performed. The coefficient vectors then yield a low-dimensional representation of the data, while the dictionary matrix contains the information on the genes that are relevant for the compression. In this paper, we apply DiL to time course transcriptomic datasets and estimate pseudotimes based on the coefficient vectors. The genes that are relevant for the dynamic changes can be derived from the dictionary matrix.

The difference of standard DiL and the suggested approach, further referred to as DiL For The Analysis Of Transcriptomic Data From Dynamic Processes (dynDLT), is, for one thing, the modification of DiL such that the dictionary is a thin-matrix and not overcomplete, which is a required modification to obtain dictionary and coefficient vectors as desired. Further, unlike in the standard DiL approach, in dynDLT the coefficient vectors need to be non-sparse. Only then can dynDLT be applied for the estimation of pseudotimes from transcriptomic datasets. Details on these steps are given in this section. We describe (1) the general DiL approach, (2) how to apply DiL to transcriptomic data, and (3) how to estimate pseudotimes from the dynDLT results.

In addition, in this section we provide details on the simulated and real-world datasets analysed, explain how we evaluate the performance of the methods and provide details on methods we compare our approach to.

### The dictionary learning approach

In DiL, for a given dataset $X=[x_{1},x_{2},...,x_{n}] \in \mathbb {R}^{p \times n}$ we want to find a dictionary matrix $D=[d_{1},d_{2},...,d_{m}] \in \mathbb {R}^{p \times m}$ and coefficient matrix $R=[r_{1},r_{2},...,r_{n}] \in \mathbb {R}^{m \times n}$, s.t.:

1 $$ x_{i} = Dr_{i} + \epsilon \quad \forall i \in 1,...,n \;,  $$

where *ε* is the reconstruction error. Typically, the dictionary matrix is chosen to be overcomplete, meaning *m*>*p*. Further, among the set of all possible solutions, the sparsest *r*_*i*_, meaning the one with the highest number of coefficients equal to 0, is desired. Together, this yields the dictionary learning problem:

2 $$ {}\mathop {\min }\limits_{D,R} \sum\limits_{i=1}^{n} \left \|{ {r_{i}} }\right \|_{0}, \quad s.t. \quad \left \|{ {X-DR} }\right \|_{F}^{2} \leq \delta \quad \forall i \in 1,...,n \;,  $$

where ∥·∥_*F*_ denotes the Frobenius norm, ∥·∥_0_ is defined as the number of non-zero elements of a vector, referred to as *l*_0_-norm, and *δ* is the reconstruction error. Additionally, ∥*d*_*i*_∥_2_=1 is typically required. The vectors *r*_*i*_ are referred to as coefficient vectors and the columns of *D* are referred to as atoms. Solving (2) is typically implemented as an iterative two-step process, where in each step it is solved for either *D* or *R*.

A formulation similar to (2), where the sparsity for each sample representation is restricted to be maximally *s*, is:

3 $$ \mathop {\min }\limits_{D,R} \left \|{ {X-DR} }\right \|_{F}^{2}, \quad s.t. \quad \left \|{ {r_{i}} }\right \|_{0} \leq s \quad \forall i \in 1,...,n \;.  $$

In this formulation, *R* is said to be the column-wise *s*-sparse.

(2) and (3) are not computationally feasible since they are non-convex problems. Ramirez et al. [19] have shown that the *l*_1_-norm is the best convex approximation for the *l*_0_-norm. Typically, DiL is implemented using the *l*_1_-norm.

### Dictionary learning for dynamic transcriptomic data

Assume we are given a transcriptomic count data matrix $Y \in \mathbb {R}^{p\times n}$, where *p* is the number of genes and *n* is the number of samples. For the computation of the dictionary and coefficient vectors, the transposed dataset *Y*^*t*^ can be considered likewise. For the atoms of *D* to be interpretable as gene-modules, it needs to be of shape *p*×*m*. Hence, we consider *Y*, because otherwise *D*∈*R*^*n*×*m*^. This approach is further referred to as *DiL For The Analysis Of Transcriptomic Data* (DLT).

In transcriptomic count data analysis, the ‘small *n* large *p*’ problem describes the problem of *n*≪*p*. Hence, using $Y \in \mathbb {R}^{p\times n}$ as the input matrix for DiL means that the dictionary is not overcomplete as $D=Y \in \mathbb {R}^{p\times n}$ would be an optimal solution to the problem. However, this solution is trivial and does not provide any new information about the data. We therefore need to choose *m*<*n* and therefore *m*≪*p*. Thus, dictionaries in DLT are a lot smaller in dimension compared to the standard DiL approach. Consequently, they are of low rank.

Certainly, by reformulating the DiL method to yield thin-matrix-dictionaries, the results differ from the traditional DiL approach. Indeed, it is not the main goal of our approach to yield a data representation that has the smallest reconstruction error to the original dataset. Rather, we aim to find those atoms, respectively gene modules, that represent the gene modules of the main underlying process. Consequently, the coefficient vectors can be interpreted as a representation of the corresponding processes. In numerical experiments (results presented in “Simulation studies” section) we show that the obtained modules are correlated with the genes exhibiting dynamic patterns. Further, the dynamic processes are well represented in the low-dimensional representation obtained from the coefficient vectors. Results for the analysis of real-world data in “Pseudotime estimation for dynamic real-world data” section support this implication. This suggests that the chosen representation is meaningful from a biological point of view.

### Estimating pseudotime based on dictionary learning

To derive pseudotimes from the DiL based analysis of dynamic transcriptomic datasets, we follow the idea of several other pseudotime approaches, e.g. [20–22], and derive pseudotimes based on the similarities of the transcriptomic profiles of the samples. This similarity is measured in the low-dimensional representation of the samples. Following this idea, the pseudotimes can be directly inferred from the values of the coefficient vector. Hence, to derive the pseudotimes we simply consider the differences in the values of the coefficient vector among all samples. Further, relevant time course genes, or marker genes, can be obtained from the dictionary. Recall, that the dictionary matrix consists of atoms, which each assign a value to each gene. Hence, the more relevant the gene is within an atom, the higher is the value of this atom entry. Thus, the highest atom values can be interpreted as marker genes for the process this atom depicts. In [18] we show that marker genes in fact depict relevant genes when DLT is applied to transcriptomic data from different phenotypes.

In numerical experiments (results presented in “Simulation studies” section) we observe that the dynamic changes in the dataset are usually captured by one atom. We therefore assume that there is one atom that captures the dynamic changes of the data. Note, that we have not yet derived a measure to determine the relevant atom. Rather, in this paper, we present evaluations for the atom that performs best among all atoms. When few sample states are known, the atom representing the dynamic module can be identified, e.g. via semi-supervised clustering.

However, assuming one atom to represent the dynamic processes requires non-zero values for all samples, as otherwise many samples would be assigned the same pseudotime based on the zero entries. Therefore, unlike in the standard DiL approach and unlike in DLT, in dynDLT the coefficient vectors need to be non-sparse. Only then can dynDLT be applied for the estimation of pseudotimes from transcriptomic datasets.

#### Using diL with existing pseudotime estimation methods

Many existing pseudotime estimation approaches construct graphs based on the low-dimensional representation of the RNA-seq dataset under study. Approaches such as PCA, ICA or t-SNE belong to the most widely used methods for dimension reduction in pseudotime estimation methods.

Apart from deriving pseudotimes from the coefficient vectors from dynDLT directly as described above, the derived low-dimensional representation can also be incorporated into existing pseudotime estimation approaches. Thereto, the method used for data compression by the respective approach, e.g. PCA, ICA, or t-SNE, is exchanged with dynDLT. Hence, the graph is learned based on the dynDLT representation. Results for such an approach are shown, by exemplary using Monocle [11], in “Pseudotime estimation for dynamic real-world data” section.

Note that for such an analysis the interpretability, which is a major benefit of dynDLT is partially lost. The reason is that the pseudotimes are no longer estimated based on the atoms, but based on the graph. This is the case for any graph-based pseudotime estimation approach, no matter which method is used to derive the low-dimensional representation. Hence, a connection to the gene modules, which are derived based on the dictionary atoms, is no longer as clear as for the pseudotimes based purely on dynDLT. This presents a major benefit of using dynDLT alone: It is a highly interpretable approach for the estimation of pseudotimes.

### Further remarks regarding the proposed diL based method

#### Positive dictionary entries

As previously described, the dictionary atoms can be interpreted as a collection of gene modules and the coefficient vectors can be interpreted as a representation of each sample based on these modules. To derive a better understanding of a gene module and the sparse representations, we restrict the dictionary to have positive values only. The values of the coefficient vectors can be positive or negative.

#### Parameters

DynDLT has two parameters: *m*, the number of dictionary atoms, and *s*, the number of non-zero entries in the representation for each sample. The dictionary is learned based on formulation (2). This means that the dictionary is learned to derive maximally sparse coefficient vectors. The idea is that this should result in atoms that represent the main gene modules.

Once the dictionary is learned, we use formulation (3) to derive the coefficient vectors. Recall, that in dynDLT we set *s*=*m*. This means that the coefficient vector is no longer sparse, but it has non-zero values for each atom per sample. The reason for this parameter choice is that pseudotime estimation coefficients are required for each sample. Accordingly, only for *s*=*m* it can be inferred how much a particular module is expressed in each sample for all atoms. A benefit of this determination is that *m* is the only parameter that has to be selected in our approach.

### Implementation and complexity

We use the Python implementation DictionaryLearning [23], which is an implementation of online dictionary learning, with default setting. It solves the problem “by efficiently minimizing at each step a quadratic surrogate function of the empirical cost over the set of constraints” [23]. This is in line with the observed times for the experiments presented.

The coefficient vectors (*r*_*i*_) are computed using orthogonal matching pursuit (OMP) [24]. A detailed complexity analysis of OMP is given in [25].

### Datasets

The proposed method for the estimation of pseudotimes of dynamic transcriptomic dataset, dynDLT, is evaluated on simulated and real-world data. In this section, the construction of the simulated data, as well as details on real-world dynamic RNA-seq datasets, are explained.

#### Simulated data

To simulate data that is similar to real-world data, simulations are based on a real-world single-cell time-course RNA-seq dataset (NCBI GEO [26] accession number: GSE87375). To prevent an influence of the time dynamics in the dataset, samples are shuffled before simulation. After an outlier detection 10,000 genes, hereinafter referred to as *g*_*or*_, and 500 samples are randomly selected. The resulting matrix is used as the baseline for the subsequent simulation.

The actual simulation is performed for a subset of the 10,000 genes, *g*_*sim*_. The simulation of values for each gene *g*_*sim*,*i*_,*i*∈[1,|*g*_*sim*_|],|*g*_*sim*_|∈[100,200,...,1000] is based on the count values of a randomly drawn gene *g*_*or*,*k*_,*k*∈[1,...,10,000]. The values are simulated to follow a particular pattern, while maintaining characteristics of the distribution of *g*_*or*,*k*_. Details on the simulation of dynamic patterns are given below.

Two types of dynamic gene expression patterns are modelled: (1) Change over time diverging from an initial state and (2) a periodic, or fluctuating change over time. The former might appear when samples are exposed to a condition over a period of time. The latter could appear in cyclic processes, for instance the cell cycle or circadian rhythms. We consider multiple parameters and additional perturbations for the construction of the simulated datasets. This way, we can track the influence of these changes on the performance in the results.

##### Simulation patterns

To simulate values for each *g*_*sim*,*i*_∈*g*_*sim*_, a gene *g*_*or*,*k*_∈*g*_*or*_ is randomly sampled from the original dataset. Datasets with either of 3 simulation patterns are each obtained by reordering the expression values of each *g*_*or*,*k*_ (compare also Fig. 8):

1. Values for all simulated genes are sorted increasingly.

**Fig. 8:**
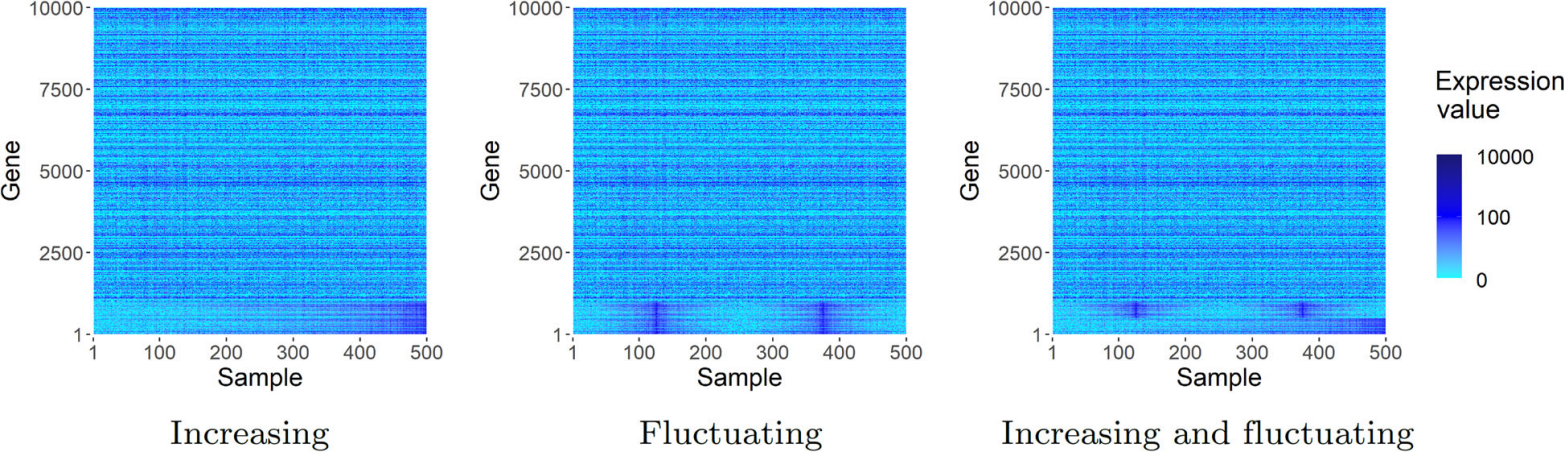
Visualisations of the 3 simulation patterns with *noise* perturbation. Shown are matrices with 1000 genes exhibiting the simulated pattern. The x-axis shows the samples ordered by simulated time. The y-axis shows the genes. The pattern is the same for all *g*_*sim*_ (simulated genes) for the *increasing* pattern and the *fluctuating* pattern, whereas for the third pattern, one half of *g*_*sim*_ follows a different subpattern than the other
2. Values for all simulated genes are sorted in a fluctuating manner: Values for each gene are partitioned into four equally large segments. The selection of values per segment is conducted randomly. Next, in the first and third segment values are sorted increasingly, while in the second and fourth segment, values are sorted decreasingly.
3. For one half of the simulated genes (|*g*_*sim*_|/2) counts are ordered increasingly, for the other half of simulated genes values are ordered fluctuating (compare pattern 2).

In several time dynamic dataset studies, e.g. [27–29] an occurrence of multiple gene expression patterns from different gene sets, as simulated in pattern 3, has been observed. Analysis of this dataset should yield insight into whether the evaluated methods can identify more than one pattern in one dataset. We refer to datasets that are simulated with pattern 3 as those with two subpatterns.

Subsequently, we add (combinations of) perturbations to the simulated gene counts: noise of different intensity and zero-counts. For the noise perturbation, for each *g*_*sim*,*i*_, random noise $\in \mathcal {N}(0,\,\sigma ^{2}(values_{g_{sim,i}}))$, or high random noise $\in \mathcal {N}(0,\,2\sigma ^{2}(values_{g_{sim,i}}))$ is added to the simulated gene counts. To maintain a real-world like dataset, which has positive integer values only, values are rounded to integers and negative values are set to their initial values before addition of noise. This yields 6 datasets for each value of |*g*_*sim*_|: For each of the 3 patterns, one with *noise* and one with *high noise* perturbation.

The previously described perturbation of added noise results in fewer zero-counts compared to the original values because many zero-values entries are overwritten with non-zero noise. To maintain a more real-world dataset, we also construct datasets in which each gene has as many zero-counts as observed in the original dataset for each *g*_*or*,*k*_. The respective samples which are assigned these zero-counts are selected randomly. Thus, in total, for each value of |*g*_*sim*_| 12 datasets are simulated.

#### Real world data

We analyse 8 real-world time course datasets from different organisms. Datasets stem from Gene Expression Omnibus [26] and ArrayExpress [30]. These include samples from bulk and single-cell experiments (see Table 1). For 2 datasets, samples stem from the one phenotype; 6 datasets contain samples from different subtypes. More details on the data are given in Additional File 1, section S2.

### Performance evaluation

To assess the performance of dynDLT for pseudotime estimation, two things can be evaluated: (1) Whether the low-dimensional representation given by the coefficient vectors preserve the time ordering of the original data (2) the overlap of marker genes derived from the dictionary atoms with the genes exhibiting the dynamic behaviour.

To determine whether the coefficient vectors derived from dynDLT represent the simulated patterns, respectively the experimental times, we evaluate the Spearman correlation of the estimated pseudotimes with the respective simulated or experimental time points. The Spearman correlation compares the ranks of the values of two vectors. For the real-world data, the values of the coefficient vector can simply be compared to the experimental time points. For the simulated data, the representative vectors for each pattern, thus the vectors the Spearman correlation are measured for, are constructed as rank vectors according to the simulation pattern (simulation pattern construction is described in “Datasets” section).

Further, for the simulated data, we evaluate whether the dictionary values corresponding to the simulated genes are high. Hence, we evaluate the performance of marker gene detection. In particular, we calculate the percentage of the simulated genes that are among the |*g*_*sim*_| highest dictionary entries. Recall, that |*g*_*sim*_| is the number of genes that are simulated to express the dynamic patterns.

For the datasets with pattern 3, hence, a set of genes with increasing counts and a set of genes with fluctuating counts over simulated time, we measure correlation and percentage for each pattern separately. We do not combine the derived values so that we can verify whether one pattern is better detected than the other.

When a method correctly identifies the imposed simulation pattern, the correlation with the representative vector as well as the percentage of correctly identified genes is high.

For the evaluation of the derived marker genes in the real-world data analysis, we consider only the atom (or component in the methods other than dynDLT) that is displaying the pseudotime. To evaluate the genes, we perform a GO-term analysis of the 500 genes with the highest absolute entries in the atom (component). It is necessary to consider absolute values, as for ICA and PCA the entries can be positive or negative. However, for our approach dynDLT and for NMF the values are positive, which allows for a better interpretation. Among all evaluated methods, the only methods suitable for such a marker gene detection are hence dynDLT, ICA, NMF, and PCA. We evaluate all GO-terms with a p-value ≤ 10^−3^.

### Comparison methods

The results from dynDLT are compared to results from independent component analysis (ICA), non-negative matrix factorisation (NMF), principal component analysis (PCA), t-distributed stochastic neighbour embedding (t-SNE), and Uniform Manifold Approximation and Projection (UMAP). Note, though, that only ICA, NMF, and PCA return a matrix decomposition. Therefore, an evaluation of correctly identified marker genes can be conducted for these methods only: Identically as performed for dynDLT, one matrix is interpreted as the matrix of gene modules and the other one is interpreted as the matrix displaying the dynamic processes. The representation of the simulated patterns is evaluated for all five comparison methods.

To interpret PCA as a method that yields a dictionary-like matrix as well as a low-dimensional representation matrix, let us firstly define the matrices resulting from PCA: Suppose $X=[x_{1}, x_{2},..., x_{n}] \in \mathbb {R}^{p \times n}$ is a column-wise mean-centred dataset. PCA then yields principal components $V = [v_{1}, v_{2},..., v_{n}] \in \mathbb {R}^{n \times n}$, with $v_{j} \in \mathbb {R}^{n}$, such that the linear transformations $Z=[z_{1}, z_{2},..., z_{n}] \in \mathbb {R}^{p \times n}$ are given by *z*_*i*_=*x*_*i*_·*v*, or equivalently:

$$\begin{array}{*{20}l} && Z & = XV\;\\ \hspace{4.0cm} & \Leftrightarrow & ZV^{-1} & = X\\\hspace{4.0cm} & \Leftrightarrow & ZV^{T} & = X\;.\hspace{4.0cm} \end{array} $$

This formulation illustrates that PCA can be directly compared to dynDLT: Recall that in dynDLT we have *DR*≈*X*. Thus, *Z* is comparable to the dictionary matrix and *V*^*T*^ to the matrix of coefficient vectors.

#### Comparison method parameters

Similar to the analysis for dynDLT, for ICA, NMF, and PCA the number of components is varied over [1,...,10]. For t-SNE and UMAP different parameters need to be considered: For t-SNE the dimensionality can be maximally 3. As for t-SNE the parameter *perplexity* has a major impact, we fix the dimensionality to 2 and evaluate the value of the *perplexity* ∈[10,20,...,100] (the default value for *perplexity* is 30). For UMAP the dimensionality is always 2. We evaluate the change of the parameter *number of neighbours* ∈[1,...,10] - the default value in the Python implementation is 5, and we thus perform a search around the default value.

For all methods the remaining parameters are set to their default values as specified in the Python library sklearn [31] (for ICA, NMF, PCA, and t-SNE), respectively umap [32] (for UMAP).

## Supplementary Information

**Additional file 1** Contains information on:
• Details on the ICA results for the simulated datasets
• Details on the real-world data evaluations:
‐ Details on the datasets that contain samples from different subtypes
‐ Details on outlier detection and normalisation for the real-world datasets
‐ Details on the merge of correlations for datasets from different subtypes

**Additional file 2** The GO-terms are shown for each of the four evaluated linear methods from whose dictionary-like matrices gene-sets can be derived. The method name is given in the second column of each table. The GO-terms are colour-coded based on whether they are (a) associated with dynamic processes in the cell, or (b) associated with the sample types or the experimental conditions. The percentage of genes (5th column) is colour-coded based on the value. Respective legends provide insight into the colour-coding.

## Data Availability

The simulated datasets are based on the NCBI Gene Expression Omnibus (GEO) [26] dataset with accession number GSE87375. The datasets analysed in the real-world data study can be found at GEO database (datasets GSE100425, GSE122380, GSE129486, GSE84712, GSE87375, GSE92652) and ArrayExpress database [30] (datasets E-MTAB-2565 and E-MTAB-6811).

## Abbreviations

DiL: Dictionary learning
GEO: Gene Expression Omnibus
ICA: Independent component analysis
MST: Minimum spanning tree
NCBI: National Center for Biotechnology Information
PCA: Principal component analysis
OMP: Orthogonal matching pursuit
(sc)RNA-seq: (Single cell) RNA-sequencing
t-SNE: t-distributed stochastic neighbour embedding
UMAP: Uniform Manifold Approximation and Projection
