# Supplementary material for: Dictionary learning allows model-free pseudotime estimation of transcriptomic data
Source: BMC Genomics. 2022 Jan 15;23:56. doi: 10.1186/s12864-021-08276-9 (PMC8760643; doi:10.1186/s12864-021-08276-9)
Supplement: Supplementary file 1 — Additional file 1 Contains information on: • Details on the ICA results for the simulated datasets • Details on the real-world data evaluations: ‐ Details on the datasets that contain samples from different subtypes ‐ Details on outlier detection and normalisation for the real-world datasets ‐ Details on the merge of correlations for datasets from different subtypes [file 12864_2021_8276_MOESM1_ESM.pdf]

Supplementary information - Dictionary learning allows model-free pseudotime estimation of transcriptomic data

S1 Simulated data evaluations

Figure S1 shows visualisations of the correlations for the ICA results.

S2 Real world data evaluations

S2.1 Data details

In the paper we present an anaylis of 8 real world time course datasets from different organisms. Datasets stem from Gene Expression Omnibus [3] and ArrayExpress [1]. These include samples from bulk and single cell experiments (for details see Table 2 in the main paper). 6 datasets contain samples from different subtypes (for details see Table S1).

To obtain datasets with the same number of samples per type, for each type, samples are randomly selected such that the number of samples per type is the same for all types and maximal given the data.

Table S1: Overview of the experimental settings of 5 real world datasets with different subtypes. Subtypes can be either explained by different cell types, different experimental conditions/treatments, or a combination of both.

**Database-ID**	**Organism**	**Cell/tissue type**	**Conditions**
GSE100425	Mus musculus	Hematopoietic stem cells of different type (short-term, long-term) and multipotent progenitors	Different age mice, (not) stimulated with inflammatory stimulus
GSE129486	Homo sapiens	Fibroblasts from individuals with rheumatoid arthritis or osteoarthritis	Stimulation with TNF or TNF + IL-17A
GSE84712	Homo sapiens	Neural progenitor cells	Lead exposure (2 different concentrations and control)
GSE87375	Mus musculus	Pancreatic Islet β-cells and α-cells	Transgenic mice (Ins1-RFP, Gcg-Cre, Rosa-RFP and Ngn3-GFP
GSE92652	Homo sapiens	Transduced hematopoietic stem cells	Lentiviral vector (LV) mediated gene correction
E-MTAB-6811	Rattus norvegicus	7 organ types (brain, cerebellum, heart, kidney, liver, ovary, testis)	-

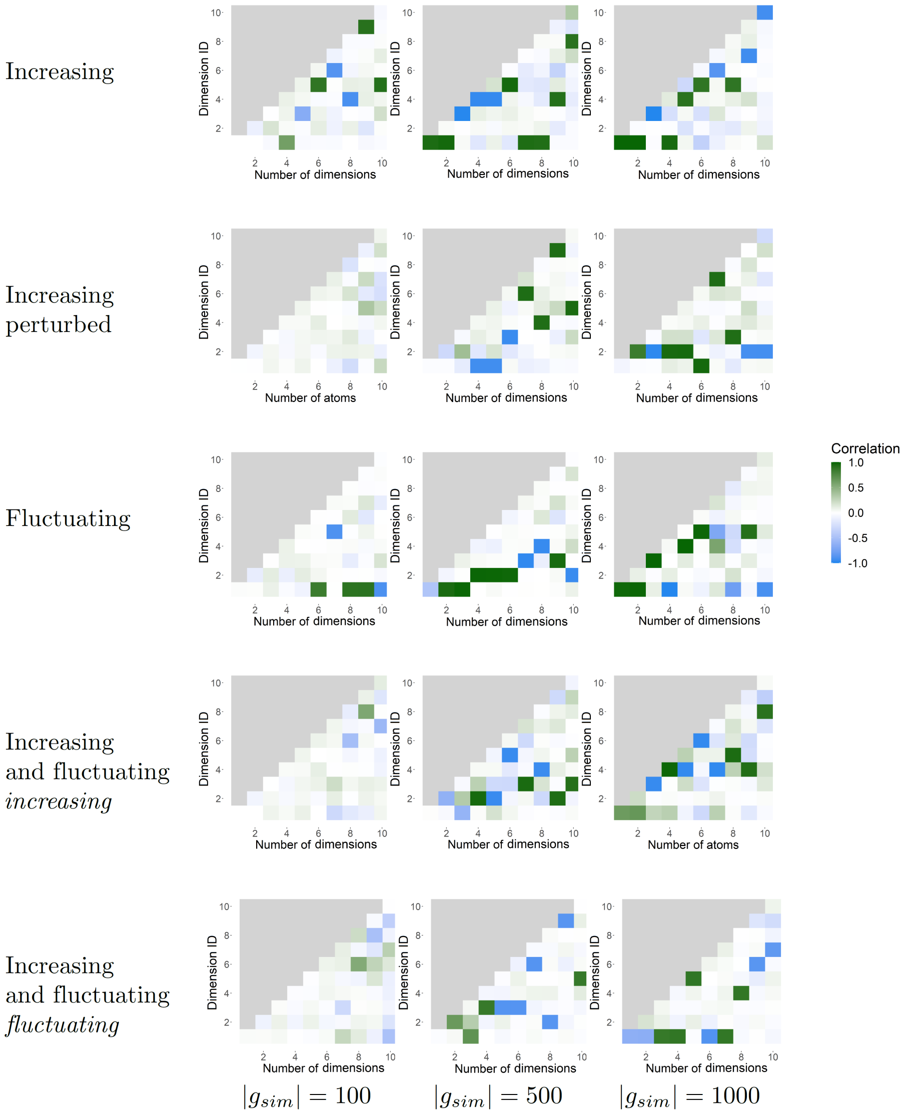

Figure S1: **Spearman correlation for the sparse code values from ICA for each parameter.** Shown are results for five simulation (sub-)pattern/ perturbation combinations in rows for three values of simulated genes exhibiting the pattern ($|g_{sim}|$) in columns. The dataset labelled ‘Increasing perturbed’ is the one with *high-noise*-and-*zero-counts* perturbation, for the other datasets only *noise* perturbation is conducted. For the dataset with an increasing and a fluctuating pattern, the correlations for each subpattern are shown separately. The respective subpattern is given in the row description in italic letters. The x-axis of each plot shows the number of dimensions and the y-axis the dimension ID. Unlike for DiL, once a high correlation is reached for a certain number of dimensions it does rarely remain high for an increase in the number of dimensions.

S2.2 Outlier detection and normalisation

Note, that this section is taken from our previous publication: [4].

In [2] Clearly et al. suggest to normalise the data by a removal of genes for which the sum of counts is $> 99.5th - percentil$ to "avoid performance statistics that are skewed by few genes with extremely high expression". We adapt this normalisation and perform the same normalisation for the samples. Additionally, to avoid for a bias of different experiments, each sample is normalised by division through the sum of all counts for this sample.

Subsequently, to provide numerical stability and allow greater interpretability of the dictionary entries, variables are centred to zero and scaled to have a standard deviation of one. This normalisation cannot be performed for NMF analysis as this results in negative and positive values. For NMF the values are rescaled to the interval $[0, 1]$.

S2.3 Merge of correlation for data with different subtypes

In the second part of the real world data evaluation, data with samples from different subtypes is analysed. To obtain one value for each type from the correlation of each subtype with the experimental times the following steps are performed:

1. For each subtype, the correlation of the experimental times and sparse code values are computed for all samples belonging to the subtype.
2. To merge all subtype values for each feature and come up with a value for the entire feature, each of these correlations is scaled by the percentage of samples that belong to the subtype.
3. The resulting values are summed for each feature.

To be considered for this assessment the following restrictions have to be fulfilled:

- For the feature to be considered
  - 1a) More than half of the subtype levels have to be measured on 3 or more time points of the entire experiment
  - 1b) The number of subtypes must not be larger than half the number of total samples
- For the subtype to be considered
  - 2a) The number of samples in the subtype is 5 or more
  - 2b) The number of time points for the subtype is 3 or more

Reasons for these restrictions are: 1a) In order to measure correlations, the data needs to consist of several time points; 1b) Only those features for which there are several subtypes with multiple samples are considered; 2a) Only those subtypes for which several measurements exist are considered; 2b) Only those subtypes which are measured on several time points are considered.

S2.4 Comparison of pseudotimes among method

To compare the methods among each other, we have performed a correlation analysis of the estimated pseudotimes among all methods evaluated. As for the evaluation of the pseudotimes when compares to the experimental times, the Spearman correlation is evaluated. Figure S2 shows the respective results. Pseudotimes are especially overlapping for the datasets in which samples stem from one phenotype (E-MTAB-2565 and GSE122380). For the other datasets a high correlation, e.g. > 0.7 appears only for some methods. One can also notice a difference between the linear methods (dynDLT, ICA, PCS, NMF) and the non-linear methods (t-SNE, UMAP) in some datasets.

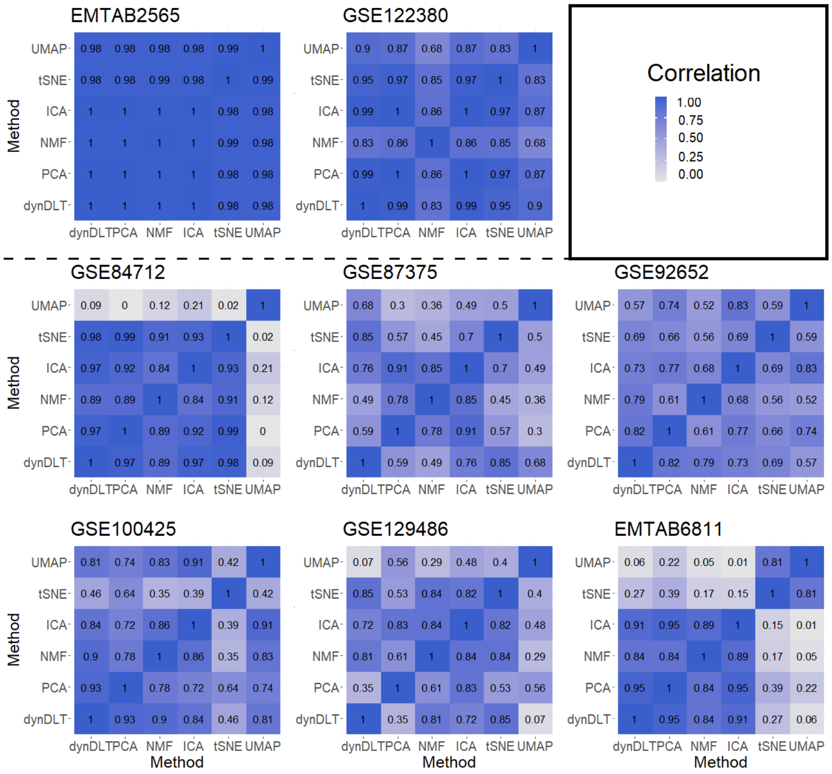

Figure S2: **Visualisation of the correlation of the estimated pseudotimes among all methods.** Shown are for each analysed dataset the correlation of the estimated pseudotimes among all methods evaluated. The correlations are highlighted based on their value. Results for the two datasets with samples from one phenotype are shown at the top. Pseudotimes are more similar for these two datasets compared to the datasets with samples from multiple phenotypes when assessed by correlation.

References

[1] A. Athar, A. Füllgrabe, N. George, H. Iqbal, L. Huerta, A. Ali, C. Snow, N. A. Fonseca, R. Petryszak, I. Papatheodorou, et al., *Arrayexpress update–from bulk to single-cell expression data*, Nucleic acids research, 47 (2019), pp. D711–D715.

[2] B. Cleary, L. Cong, A. Cheung, E. S. Lander, and A. Regev, *Efficient generation of transcriptomic profiles by random composite measurements*, Cell, 171 (2017), pp. 1424–1436.

[3] R. Edgar, M. Domrachev, and A. E. Lash, *Gene expression omnibus: Ncbi gene expression and hybridization array data repository*, Nucleic acids research, 30 (2002), pp. 207–210.

[4] M. Rams and T. Conrad, *Dictionary learning for transcriptomics data reveals type-specific gene modules in a multi-class setting*, it-Information Technology, 1 (2020).
