# Supplementary material for: Dictionary learning allows model-free pseudotime estimation of transcriptomic data
Source: BMC Genomics. 2022 Jan 15;23:56. doi: 10.1186/s12864-021-08276-9 (PMC8760643; doi:10.1186/s12864-021-08276-9)
Supplement: Supplementary file 2 — Additional file 2 The GO-terms are shown for each of the four evaluated linear methods from whose dictionary-like matrices gene-sets can be derived. The method name is given in the second column of each table. The GO-terms are colour-coded based on whether they are (a) associated with dynamic processes in the cell, or (b) associated with the sample types or the experimental conditions. The percentage of genes (5th column) is colour-coded based on the value. Respective legends provide insight into the colour-coding. [file 12864_2021_8276_MOESM2_ESM.pdf]

GO-term evaluation for the real-world data analysis

The tables on the following pages show the results for the GO-term analysis from the real-world data analysis. On each page, the results for one of the eight datasets are shown.

In six columns, the dataset name, method name, GO-term, GO-term p-value, the number of genes in the gene-set overlapping with the genes from the particular GO-term, and the percentage these genes make among all genes associated with that GO-term, are listed.

The GO-terms are shown for each of the methods, from whose dictionary-like matrices gene-sets can be derived. The method name is given in the second column of each table.

The GO-terms are colour-coded based on whether they are associated with (a) dynamic cell processes, or (b) the sample types, respectively the experimental conditions. The percentage of genes (5th column) is colour-coded based on the value. Respective legends provide insight into the colour-coding.

Dataset	Method	GO term	P-Value	#Genes	%Overlap
GSE100425	dynDLT	oxidation-reduction process	5.21E-08	40	8.6
GSE100425	dynDLT	purine nucleotide biosynthetic process	3.41E-06	7	1.51
GSE100425	dynDLT	'de novo' IMP biosynthetic process	3.45E-06	5	1.08
GSE100425	dynDLT	rRNA processing	0.000024	13	2.8
GSE100425	dynDLT	tricarboxylic acid cycle	3.53E-05	7	1.51
GSE100425	dynDLT	response to iron ion	4.71E-05	6	1.29
GSE100425	dynDLT	mitochondrion organization	7.56E-05	10	2.15
GSE100425	dynDLT	metabolic process	0.000113	25	5.38
GSE100425	dynDLT	nucleotide metabolic process	0.000282	6	1.29
GSE100425	dynDLT	pseudouridine synthesis	0.000351	5	1.08
GSE100425	dynDLT	ribonucleoside monophosphate biosynthetic process	0.000355	4	0.86
GSE100425	dynDLT	fatty acid beta-oxidation	0.000397	7	1.51
GSE100425	dynDLT	cell proliferation	0.000416	15	3.23
GSE100425	dynDLT	purine nucleotide metabolic process	0.000558	4	0.86
GSE100425	dynDLT	pyrimidine nucleotide metabolic process	0.000558	4	0.86
GSE100425	dynDLT	definitive hemopoiesis	0.00057	5	1.08
GSE100425	dynDLT	UTP biosynthetic process	0.000823	4	0.86
GSE100425	ICA	inflammatory response	9E-11	33	6.88
GSE100425	ICA	intrinsic apoptotic signaling pathway in response to DNA damage	5.05E-09	13	2.71
GSE100425	ICA	regulation of apoptotic process	1.84E-08	22	4.58
GSE100425	ICA	response to lipopolysaccharide	3.65E-07	20	4.17
GSE100425	ICA	immune response	9.31E-07	23	4.79
GSE100425	ICA	oxidation-reduction process	3.95E-06	38	7.92
GSE100425	ICA	cell proliferation	2.89E-05	18	3.75
GSE100425	ICA	release of cytochrome c from mitochondria	3.05E-05	7	1.46
GSE100425	ICA	isoprenoid biosynthetic process	4.05E-05	6	1.25
GSE100425	ICA	regulation of cell proliferation	4.29E-05	18	3.75
GSE100425	ICA	DNA replication	4.66E-05	13	2.71
GSE100425	ICA	negative regulation of apoptotic process	5.99E-05	31	6.46
GSE100425	ICA	immune system process	6.93E-05	24	5
GSE100425	ICA	positive regulation of NF-kappaB transcription factor activity	7.05E-05	12	2.5
GSE100425	ICA	positive regulation of I-kappaB kinase/NF-kappaB signaling	7.32E-05	14	2.92
GSE100425	ICA	response to drug	9.22E-05	22	4.58
GSE100425	ICA	purine nucleotide biosynthetic process	9.56E-05	6	1.25
GSE100425	ICA	response to oxidative stress	9.99E-05	13	2.71
GSE100425	ICA	cellular response to DNA damage stimulus	0.000103	25	5.21
GSE100425	ICA	cholesterol biosynthetic process	0.000106	7	1.46
GSE100425	ICA	cell chemotaxis	0.000114	10	2.08
GSE100425	ICA	cellular response to interleukin-1	0.000139	10	2.08
GSE100425	ICA	cellular response to lipopolysaccharide	0.000192	16	3.33
GSE100425	ICA	response to toxic substance	0.000242	10	2.08
GSE100425	ICA	'de novo' IMP biosynthetic process	0.000271	4	0.83
GSE100425	ICA	innate immune response	0.000343	23	4.79
GSE100425	ICA	chemokine-mediated signaling pathway	0.000357	8	1.67
GSE100425	ICA	positive regulation of peptidyl-serine phosphorylation	0.000427	9	1.88
GSE100425	ICA	sterol biosynthetic process	0.000433	6	1.25
GSE100425	ICA	protein tetramerization	0.000438	7	1.46
GSE100425	ICA	metabolic process	0.000447	25	5.21
GSE100425	ICA	extrinsic apoptotic signaling pathway in absence of ligand	0.000501	7	1.46
GSE100425	ICA	positive regulation of apoptotic process	0.000591	20	4.17
GSE100425	ICA	negative regulation of reactive oxygen species metabolic process	0.000611	6	1.25
GSE100425	ICA	defense response to protozoan	0.000719	6	1.25
GSE100425	ICA	pyrimidine nucleotide metabolic process	0.000732	4	0.83
GSE100425	ICA	lipopolysaccharide-mediated signaling pathway	0.00084	6	1.25
GSE100425	ICA	negative regulation of viral genome replication	0.000976	6	1.25
GSE100425	ICA	response to virus	0.001	9	1.88
GSE100425	NMF	translation	2.3E-29	61	12.79
GSE100425	NMF	formation of translation preinitiation complex	5.71E-15	14	2.94
GSE100425	NMF	translational initiation	1.04E-14	18	3.77
GSE100425	NMF	cell-cell adhesion	3.04E-13	28	5.87
GSE100425	NMF	regulation of translational initiation	5.24E-13	14	2.94
GSE100425	NMF	protein folding	1.11E-12	23	4.82
GSE100425	NMF	protein stabilization	3.73E-09	19	3.98
GSE100425	NMF	positive regulation of protein localization to Cajal body	6.77E-09	7	1.47
GSE100425	NMF	cytoplasmic translation	1.16E-07	10	2.1
GSE100425	NMF	RNA splicing	2.23E-07	23	4.82
GSE100425	NMF	negative regulation of apoptotic process	4.01E-07	37	7.76
GSE100425	NMF	positive regulation of telomerase RNA localization to Cajal body	1.04E-06	7	1.47
GSE100425	NMF	positive regulation of establishment of protein localization to telomere	1.17E-06	6	1.26
GSE100425	NMF	platelet aggregation	3.93E-06	9	1.89
GSE100425	NMF	antigen processing and presentation of exogenous peptide antigen via MHC class I, TAP-dependent	7.74E-06	8	1.68
GSE100425	NMF	toxin transport	2.29E-05	8	1.68
GSE100425	NMF	mRNA processing	2.57E-05	23	4.82
GSE100425	NMF	binding of sperm to zona pellucida	2.78E-05	8	1.68
GSE100425	NMF	DNA replication	0.000067	13	2.73
GSE100425	NMF	DNA unwinding involved in DNA replication	7.49E-05	5	1.05
GSE100425	NMF	positive regulation of telomere maintenance via telomerase	0.000131	7	1.47
GSE100425	NMF	ribosomal small subunit assembly	0.000147	6	1.26
GSE100425	NMF	response to drug	0.000157	22	4.61
GSE100425	NMF	cell redox homeostasis	0.000224	9	1.89
GSE100425	NMF	glycolytic process	0.000258	7	1.47
GSE100425	NMF	DNA replication initiation	0.000288	6	1.26
GSE100425	NMF	IRES-dependent viral translational initiation	0.00052	4	0.84
GSE100425	NMF	positive regulation of translation	0.000558	8	1.68
GSE100425	NMF	tricarboxylic acid cycle	0.000726	6	1.26
GSE100425	NMF	negative regulation of cell death	0.000924	9	1.89
GSE100425	NMF	actin cytoskeleton organization	0.000976	12	2.52
GSE100425	NMF	cell cycle	0.00098	30	6.29
GSE100425	PCA	cell cycle	1.46E-14	51	10.83
GSE100425	PCA	cell division	3.38E-12	36	7.64
GSE100425	PCA	mitotic nuclear division	8.87E-11	29	6.16
GSE100425	PCA	DNA replication	5.89E-10	19	4.03
GSE100425	PCA	DNA replication initiation	0.000204	6	1.27
GSE100425	PCA	DNA-dependent DNA replication	0.000434	5	1.06
GSE100425	PCA	response to cytokine	0.000617	9	1.91
GSE100425	PCA	heme biosynthetic process	0.000874	5	1.06

GO TERM COLOUR LEGEND

- development
- differentation
- proliferation
- cell cycle
- mitosis
- mitotic
- G1/S
- G2/M
- circadian
- genesis

Dataset/subtype conditioned highlighting

Dataset/subtype conditions highlighted

Immune system/ hematopoesis related

%Overlap Legend

20

10

0

Dataset	Method	GO term	P-Value	#Genes	%Overlap
GSE122380	dynDLT	cilium morphogenesis	5.94E-07	16	3.2
GSE122380	dynDLT	transcription, DNA-templated	3.17E-06	77	15.4
GSE122380	dynDLT	cilium assembly	3.03E-05	13	2.6
GSE122380	dynDLT	regulation of transcription, DNA-templated	3.79E-05	60	12
GSE122380	dynDLT	embryonic skeletal system morphogenesis	2.51E-04	7	1.4
GSE122380	dynDLT	anterior/posterior pattern specification	5.14E-04	9	1.8
GSE122380	dynDLT	mRNA splice site selection	5.26E-04	5	1
GSE122380	dynDLT	inner ear receptor stereocilium organization	8.26E-04	5	1
GSE122380	ICA	cell division	9.60E-20	47	9.4
GSE122380	ICA	DNA replication	5.35E-17	30	6
GSE122380	ICA	mitotic nuclear division	8.21E-11	29	5.8
GSE122380	ICA	G1/S transition of mitotic cell cycle	1.44E-10	19	3.8
GSE122380	ICA	DNA repair	1.39E-08	25	5
GSE122380	ICA	DNA replication initiation	8.81E-08	10	2
GSE122380	ICA	mitotic nuclear envelope disassembly	1.69E-06	10	2
GSE122380	ICA	sister chromatid cohesion	2.89E-06	14	2.8
GSE122380	ICA	double-strand break repair	7.82E-06	11	2.2
GSE122380	ICA	G2/M transition of mitotic cell cycle	1.47E-05	15	3
GSE122380	ICA	tRNA export from nucleus	1.54E-05	8	1.6
GSE122380	ICA	anaphase-promoting complex-dependent catabolic process	3.95E-05	11	2.2
GSE122380	ICA	cellular response to DNA damage stimulus	1.23E-04	17	3.4
GSE122380	ICA	viral process	1.27E-04	21	4.2
GSE122380	ICA	mitotic spindle assembly checkpoint	1.35E-04	6	1.2
GSE122380	ICA	telomere maintenance via recombination	1.59E-04	7	1.4
GSE122380	ICA	regulation of glucose transport	1.90E-04	7	1.4
GSE122380	ICA	cell cycle	2.00E-04	17	3.4
GSE122380	ICA	protein sumoylation	2.53E-04	12	2.4
GSE122380	ICA	base-excision repair	2.66E-04	7	1.4
GSE122380	ICA	cell proliferation	2.76E-04	23	4.6
GSE122380	ICA	'de novo' IMP biosynthetic process	3.37E-04	4	0.8
GSE122380	ICA	intracellular transport of virus	3.46E-04	8	1.6
GSE122380	ICA	strand displacement	5.04E-04	6	1.2
GSE122380	ICA	protein ubiquitination	5.43E-04	22	4.4
GSE122380	ICA	spindle organization	6.59E-04	5	1
GSE122380	ICA	regulation of cellular response to heat	7.58E-04	9	1.8
GSE122380	ICA	positive regulation of ubiquitin-protein ligase activity involved in regulation of mitotic cell cycle transition	8.28E-04	9	1.8
GSE122380	ICA	DNA replication checkpoint	9.07E-04	4	0.8
GSE122380	ICA	DNA duplex unwinding	9.50E-04	7	1.4
GSE122380	NMF	cell-cell adhesion	2.02E-17	40	8
GSE122380	NMF	translational initiation	4.84E-13	25	5
GSE122380	NMF	SRP-dependent cotranslational protein targeting to membrane	9.71E-12	20	4
GSE122380	NMF	nuclear-transcribed mRNA catabolic process, nonsense-mediated decay	1.22E-11	22	4.4
GSE122380	NMF	viral transcription	1.76E-09	19	3.8
GSE122380	NMF	cell adhesion	1.95E-09	39	7.8
GSE122380	NMF	sarcomere organization	5.52E-08	10	2
GSE122380	NMF	muscle filament sliding	5.79E-08	11	2.2
GSE122380	NMF	extracellular matrix organization	1.30E-07	22	4.4
GSE122380	NMF	osteoblast differentiation	1.77E-07	16	3.2
GSE122380	NMF	muscle contraction	2.59E-07	16	3.2
GSE122380	NMF	heart development	8.37E-07	20	4
GSE122380	NMF	response to hypoxia	1.45E-06	19	3.8
GSE122380	NMF	axon guidance	2.11E-06	18	3.6
GSE122380	NMF	actin filament organization	4.39E-06	12	2.4
GSE122380	NMF	cell migration	6.17E-06	18	3.6
GSE122380	NMF	cytoskeletal anchoring at plasma membrane	6.48E-06	6	1.2
GSE122380	NMF	translation	8.34E-06	22	4.4
GSE122380	NMF	regulation of cardiac muscle contraction by regulation of the release of sequestered calcium ion	8.82E-06	7	1.4
GSE122380	NMF	platelet degranulation	2.81E-05	13	2.6
GSE122380	NMF	cardiac muscle contraction	2.90E-05	9	1.8
GSE122380	NMF	cell growth involved in cardiac muscle cell development	3.75E-05	5	1
GSE122380	NMF	cytoskeleton organization	4.32E-05	16	3.2
GSE122380	NMF	positive regulation of gene expression	4.55E-05	21	4.2
GSE122380	NMF	adherens junction organization	6.06E-05	8	1.6
GSE122380	NMF	canonical glycolysis	6.35E-05	7	1.4
GSE122380	NMF	endodermal cell differentiation	7.97E-05	7	1.4
GSE122380	NMF	erythrocyte differentiation	1.02E-04	8	1.6
GSE122380	NMF	rRNA processing	1.03E-04	18	3.6
GSE122380	NMF	regulation of ryanodine-sensitive calcium-release channel activity	1.36E-04	6	1.2
GSE122380	NMF	regulation of cardiac conduction	1.46E-04	9	1.8
GSE122380	NMF	vasculogenesis	1.46E-04	9	1.8
GSE122380	NMF	mRNA splicing, via spliceosome	1.61E-04	18	3.6
GSE122380	NMF	heart morphogenesis	2.17E-04	7	1.4
GSE122380	NMF	RNA splicing	2.23E-04	15	3
GSE122380	NMF	regulation of heart rate	2.59E-04	7	1.4
GSE122380	NMF	actin cytoskeleton organization	2.70E-04	13	2.6
GSE122380	NMF	positive regulation of apoptotic process	2.86E-04	21	4.2
GSE122380	NMF	epithelial to mesenchymal transition	3.07E-04	7	1.4
GSE122380	NMF	glycolytic process	3.07E-04	7	1.4
GSE122380	NMF	atrial septum morphogenesis	3.43E-04	5	1
GSE122380	NMF	viral entry into host cell	3.66E-04	10	2
GSE122380	NMF	regulation of translational initiation	4.25E-04	7	1.4
GSE122380	NMF	angiogenesis	5.16E-04	17	3.4
GSE122380	NMF	embryo development	5.74E-04	7	1.4
GSE122380	NMF	protein targeting	6.63E-04	7	1.4
GSE122380	NMF	chorio-allantoic fusion	6.84E-04	4	0.8
GSE122380	NMF	viral process	7.24E-04	20	4
GSE122380	NMF	response to muscle stretch	8.17E-04	5	1
GSE122380	NMF	neuromuscular junction development	9.34E-04	6	1.2
GSE122380	PCA	cell division	9.60E-20	47	9.4
GSE122380	PCA	DNA replication	5.35E-17	30	6
GSE122380	PCA	mitotic nuclear division	8.21E-11	29	5.8
GSE122380	PCA	G1/S transition of mitotic cell cycle	1.44E-10	19	3.8
GSE122380	PCA	DNA repair	1.39E-08	25	5
GSE122380	PCA	DNA replication initiation	8.81E-08	10	2
GSE122380	PCA	mitotic nuclear envelope disassembly	1.69E-06	10	2
GSE122380	PCA	sister chromatid cohesion	2.89E-06	14	2.8
GSE122380	PCA	double-strand break repair	7.82E-06	11	2.2
GSE122380	PCA	G2/M transition of mitotic cell cycle	1.47E-05	15	3
GSE122380	PCA	tRNA export from nucleus	1.54E-05	8	1.6
GSE122380	PCA	anaphase-promoting complex-dependent catabolic process	3.95E-05	11	2.2
GSE122380	PCA	cellular response to DNA damage stimulus	1.23E-04	17	3.4
GSE122380	PCA	viral process	1.27E-04	21	4.2
GSE122380	PCA	mitotic spindle assembly checkpoint	1.35E-04	6	1.2
GSE122380	PCA	telomere maintenance via recombination	1.59E-04	7	1.4
GSE122380	PCA	regulation of glucose transport	1.90E-04	7	1.4
GSE122380	PCA	cell cycle	2.00E-04	17	3.4
GSE122380	PCA	protein sumoylation	2.53E-04	12	2.4
GSE122380	PCA	base-excision repair	2.66E-04	7	1.4
GSE122380	PCA	cell proliferation	2.76E-04	23	4.6
GSE122380	PCA	'de novo' IMP biosynthetic process	3.37E-04	4	0.8
GSE122380	PCA	intracellular transport of virus	3.46E-04	8	1.6
GSE122380	PCA	strand displacement	5.04E-04	6	1.2
GSE122380	PCA	protein ubiquitination	5.43E-04	22	4.4
GSE122380	PCA	spindle organization	6.59E-04	5	1
GSE122380	PCA	regulation of cellular response to heat	7.58E-04	9	1.8
GSE122380	PCA	positive regulation of ubiquitin-protein ligase activity involved in regulation of mitotic cell cycle transition	8.28E-04	9	1.8
GSE122380	PCA	DNA replication checkpoint	9.07E-04	4	0.8
GSE122380	PCA	DNA duplex unwinding	9.50E-04	7	1.4

GO TERM COLOUR LEGEND

- development
- differentiation
- proliferation
- cell cycle
- mitosis
- mitotic
- G1/S
- G2/M
- circadian
- genesis

Dataset/subtype conditioned highlighting

Dataset/subtype conditions highlighted

Stem/ heart/ cardiac/ muscle cell related

%Overlap Legend

20

10

0

Dataset	Method	GO term	P-Value	#Genes	%Overlap
GSE129486	dynDLT	lung alveolus development	1.72E-05	8	1.6
GSE129486	dynDLT	platelet degranulation	5.21E-05	12	2.4
GSE129486	dynDLT	branching involved in prostate gland morphogenesis	6.06E-05	4	0.8
GSE129486	dynDLT	skeletal system development	1.67E-04	13	2.6
GSE129486	dynDLT	odontogenesis	4.84E-04	6	1.2
GSE129486	dynDLT	response to wounding	9.51E-04	8	1.6
GSE129486	ICA	actin cytoskeleton organization	4.49E-05	14	2.8
GSE129486	ICA	cellular response to hypoxia	4.95E-05	12	2.4
GSE129486	ICA	signal transduction	8.73E-05	54	10.8
GSE129486	ICA	angiogenesis	1.06E-04	18	3.6
GSE129486	ICA	positive regulation of cell proliferation	7.75E-04	26	5.2
GSE129486	NMF	SRP-dependent cotranslational protein targeting to membrane	4.00E-48	48	9.6
GSE129486	NMF	translational initiation	8.50E-47	54	10.8
GSE129486	NMF	nuclear-transcribed mRNA catabolic process, nonsense-mediated decay	6.23E-38	45	9
GSE129486	NMF	viral transcription	6.44E-38	44	8.8
GSE129486	NMF	translation	1.82E-27	50	10
GSE129486	NMF	rRNA processing	7.08E-26	45	9
GSE129486	NMF	cell-cell adhesion	3.61E-18	41	8.2
GSE129486	NMF	protein folding	6.27E-09	23	4.6
GSE129486	NMF	positive regulation of protein localization to Cajal body	1.21E-08	7	1.4
GSE129486	NMF	cytoplasmic translation	1.29E-08	10	2
GSE129486	NMF	regulation of translational initiation	4.53E-07	10	2
GSE129486	NMF	ER to Golgi vesicle-mediated transport	5.30E-07	19	3.8
GSE129486	NMF	movement of cell or subcellular component	7.07E-07	14	2.8
GSE129486	NMF	positive regulation of telomerase RNA localization to Cajal body	1.83E-06	7	1.4
GSE129486	NMF	positive regulation of establishment of protein localization to telomere	1.89E-06	6	1.2
GSE129486	NMF	cell adhesion	1.98E-06	33	6.6
GSE129486	NMF	formation of translation preinitiation complex	2.08E-06	8	1.6
GSE129486	NMF	ribosomal small subunit biogenesis	2.87E-06	7	1.4
GSE129486	NMF	protein stabilization	5.99E-06	16	3.2
GSE129486	NMF	ribosomal small subunit assembly	9.04E-06	7	1.4
GSE129486	NMF	Wnt signaling pathway, planar cell polarity pathway	9.18E-06	13	2.6
GSE129486	NMF	retrograde vesicle-mediated transport, Golgi to ER	1.65E-05	12	2.4
GSE129486	NMF	osteoblast differentiation	3.23E-05	13	2.6
GSE129486	NMF	negative regulation of apoptotic process	3.24E-05	30	6
GSE129486	NMF	COPII vesicle coating	4.50E-05	10	2
GSE129486	NMF	proteolysis involved in cellular protein catabolic process	4.86E-05	9	1.8
GSE129486	NMF	toxin transport	5.17E-05	8	1.6
GSE129486	NMF	negative regulation of canonical Wnt signaling pathway	5.24E-05	16	3.2
GSE129486	NMF	viral process	1.04E-04	22	4.4
GSE129486	NMF	protein N-linked glycosylation via asparagine	1.05E-04	8	1.6
GSE129486	NMF	intracellular protein transport	1.15E-04	19	3.8
GSE129486	NMF	antigen processing and presentation of peptide antigen via MHC class I	1.53E-04	7	1.4
GSE129486	NMF	positive regulation of substrate adhesion-dependent cell spreading	2.22E-04	7	1.4
GSE129486	NMF	positive regulation of telomere maintenance via telomerase	2.22E-04	7	1.4
GSE129486	NMF	barbed-end actin filament capping	4.77E-04	5	1
GSE129486	NMF	platelet degranulation	5.99E-04	11	2.2
GSE129486	NMF	ephrin receptor signaling pathway	6.46E-04	10	2
GSE129486	NMF	response to reactive oxygen species	6.78E-04	7	1.4
GSE129486	NMF	tRNA aminoacylation for protein translation	7.79E-04	7	1.4
GSE129486	NMF	regulation of mitochondrial membrane potential	9.52E-04	6	1.2
GSE129486	PCA	defense response to virus	9.66E-08	20	4
GSE129486	PCA	type I interferon signaling pathway	9.37E-07	12	2.4
GSE129486	PCA	actin cytoskeleton organization	1.03E-05	15	3
GSE129486	PCA	response to virus	3.88E-05	13	2.6
GSE129486	PCA	negative regulation of viral genome replication	8.21E-05	8	1.6
GSE129486	PCA	negative regulation of apoptotic process	2.52E-04	27	5.4
GSE129486	PCA	proteolysis involved in cellular protein catabolic process	2.70E-04	8	1.6

GO TERM COLOUR LEGEND

- development
- differentiation
- proliferation
- cell cycle
- mitosis
- mitotic
- G1/S
- G2/M
- circadian
- genesis

Dataset/subtype conditioned highlighting

Dataset/subtype conditions highlighted

Cell migration/ cell adhesion/ connective tissue related

%Overlap Legend

20

10

0

Dataset	Method	GO term	P-Value	#Genes	%Overlap
GSE84712	dynDLT	neurotransmitter secretion	2.47E-08	12	2.6
GSE84712	dynDLT	positive regulation of GTPase activity	2.57E-07	36	7.81
GSE84712	dynDLT	chemical synaptic transmission	3.18E-07	22	4.77
GSE84712	dynDLT	nervous system development	4.21E-07	24	5.21
GSE84712	dynDLT	synapse assembly	1.60E-06	11	2.39
GSE84712	dynDLT	long-term synaptic potentiation	2.53E-06	9	1.95
GSE84712	dynDLT	glutamate secretion	3.17E-06	8	1.74
GSE84712	dynDLT	positive regulation of excitatory postsynaptic potential	5.14E-06	7	1.52
GSE84712	dynDLT	vesicle fusion	9.89E-06	10	2.17
GSE84712	dynDLT	protein localization to plasma membrane	1.71E-05	10	2.17
GSE84712	dynDLT	positive regulation of calcium ion-dependent exocytosis	2.63E-05	6	1.3
GSE84712	dynDLT	adult behavior	2.70E-05	7	1.52
GSE84712	dynDLT	calcium ion-regulated exocytosis of neurotransmitter	3.29E-05	8	1.74
GSE84712	dynDLT	social behavior	1.31E-04	8	1.74
GSE84712	dynDLT	gamma-aminobutyric acid signaling pathway	1.41E-04	6	1.3
GSE84712	dynDLT	regulation of calcium ion-dependent exocytosis	1.59E-04	7	1.52
GSE84712	dynDLT	regulation of potassium ion transmembrane transport	3.51E-04	5	1.08
GSE84712	dynDLT	learning	3.91E-04	8	1.74
GSE84712	dynDLT	neuron cell-cell adhesion	4.60E-04	5	1.08
GSE84712	dynDLT	ion transmembrane transport	5.31E-04	15	3.25
GSE84712	dynDLT	potassium ion transmembrane transport	6.35E-04	11	2.39
GSE84712	dynDLT	inositol phosphate metabolic process	8.27E-04	7	1.52
GSE84712	dynDLT	positive regulation of synaptic transmission, glutamatergic	9.26E-04	5	1.08
GSE84712	ICA	mRNA splicing, via spliceosome	2.44E-10	26	5.65
GSE84712	ICA	neurotransmitter secretion	3.77E-07	11	2.39
GSE84712	ICA	intracellular signal transduction	6.43E-05	25	5.43
GSE84712	ICA	RNA splicing	2.40E-04	14	3.04
GSE84712	ICA	viral transcription	4.41E-04	11	2.39
GSE84712	ICA	nervous system development	7.84E-04	18	3.91
GSE84712	ICA	apoptotic process	8.55E-04	28	6.09
GSE84712	ICA	protein phosphorylation	9.52E-04	24	5.22
GSE84712	NMF	translational initiation	3.26E-83	76	16.2
GSE84712	NMF	SRP-dependent cotranslational protein targeting to membrane	5.84E-82	66	14.07
GSE84712	NMF	nuclear-transcribed mRNA catabolic process, nonsense-mediated decay	2.19E-75	68	14.5
GSE84712	NMF	viral transcription	8.15E-71	64	13.65
GSE84712	NMF	translation	2.40E-52	71	15.14
GSE84712	NMF	rRNA processing	1.78E-51	66	14.07
GSE84712	NMF	cell-cell adhesion	2.23E-18	40	8.53
GSE84712	NMF	cytoplasmic translation	8.28E-15	14	2.99
GSE84712	NMF	mRNA splicing, via spliceosome	2.03E-14	32	6.82
GSE84712	NMF	gene expression	1.14E-11	15	3.2
GSE84712	NMF	ATP-dependent chromatin remodeling	2.98E-09	10	2.13
GSE84712	NMF	regulation of translational initiation	1.78E-08	11	2.35
GSE84712	NMF	nucleosome disassembly	1.20E-07	8	1.71
GSE84712	NMF	ribosomal small subunit assembly	2.96E-07	8	1.71
GSE84712	NMF	osteoblast differentiation	5.00E-07	15	3.2
GSE84712	NMF	formation of translation preinitiation complex	1.32E-06	8	1.71
GSE84712	NMF	ribosomal small subunit biogenesis	1.93E-06	7	1.49
GSE84712	NMF	regulation of mRNA stability	2.68E-06	14	2.99
GSE84712	NMF	G2/M transition of mitotic cell cycle	2.86E-06	16	3.41
GSE84712	NMF	Wnt signaling pathway, planar cell polarity pathway	4.58E-06	13	2.77
GSE84712	NMF	chromatin remodeling	1.38E-05	12	2.56
GSE84712	NMF	positive regulation of protein localization to Cajal body	2.91E-05	5	1.07
GSE84712	NMF	establishment of integrated proviral latency	2.91E-05	5	1.07
GSE84712	NMF	positive regulation of transcription from RNA polymerase II promoter	3.90E-05	48	10.23
GSE84712	NMF	positive regulation of viral genome replication	4.44E-05	7	1.49
GSE84712	NMF	cholesterol biosynthetic process	4.84E-05	8	1.71
GSE84712	NMF	mRNA processing	7.08E-05	16	3.41
GSE84712	NMF	protein folding	7.55E-05	16	3.41
GSE84712	NMF	cell proliferation	9.49E-05	24	5.12
GSE84712	NMF	negative regulation of translation	1.21E-04	9	1.92
GSE84712	NMF	ribosomal large subunit assembly	1.68E-04	6	1.28
GSE84712	NMF	CRD-mediated mRNA stabilization	1.68E-04	4	0.85
GSE84712	NMF	positive regulation of transcription, DNA-templated	2.06E-04	29	6.18
GSE84712	NMF	RNA processing	2.15E-04	11	2.35
GSE84712	NMF	regulation of circadian rhythm	2.57E-04	8	1.71
GSE84712	NMF	axon guidance	2.71E-04	14	2.99
GSE84712	NMF	toxin transport	3.01E-04	7	1.49
GSE84712	NMF	DNA damage response, detection of DNA damage	3.01E-04	7	1.49
GSE84712	NMF	positive regulation of muscle cell differentiation	3.29E-04	6	1.28
GSE84712	NMF	viral process	3.30E-04	20	4.26
GSE84712	NMF	negative regulation of apoptotic process	3.80E-04	26	5.54
GSE84712	NMF	ribosomal large subunit biogenesis	4.03E-04	6	1.28
GSE84712	NMF	cell adhesion	4.33E-04	26	5.54
GSE84712	NMF	positive regulation of telomerase RNA localization to Cajal body	4.92E-04	5	1.07
GSE84712	NMF	actin cytoskeleton organization	5.91E-04	12	2.56
GSE84712	NMF	antigen processing and presentation of exogenous peptide antigen via MHC class II	6.49E-04	10	2.13
GSE84712	NMF	positive regulation of DNA binding	6.99E-04	6	1.28
GSE84712	NMF	covalent chromatin modification	7.33E-04	11	2.35
GSE84712	NMF	protein import into nucleus	7.37E-04	8	1.71
GSE84712	NMF	protein stabilization	8.63E-04	12	2.56
GSE84712	NMF	ribosomal protein import into nucleus	8.89E-04	4	0.85
GSE84712	NMF	positive regulation of apoptotic process	9.17E-04	19	4.05
GSE84712	PCA	mRNA splicing, via spliceosome	7.63E-17	34	7.41
GSE84712	PCA	mRNA export from nucleus	3.39E-10	18	3.92
GSE84712	PCA	RNA splicing	1.53E-07	19	4.14
GSE84712	PCA	chromatin remodeling	1.75E-07	14	3.05
GSE84712	PCA	termination of RNA polymerase II transcription	3.44E-06	11	2.4
GSE84712	PCA	viral process	5.37E-06	23	5.01
GSE84712	PCA	RNA export from nucleus	7.19E-06	10	2.18
GSE84712	PCA	gene expression	2.03E-05	9	1.96
GSE84712	PCA	mRNA 3'-end processing	2.76E-05	9	1.96
GSE84712	PCA	protein sumoylation	3.19E-05	13	2.83
GSE84712	PCA	intracellular transport of virus	3.21E-05	9	1.96
GSE84712	PCA	mRNA processing	3.84E-05	16	3.49
GSE84712	PCA	tRNA export from nucleus	1.14E-04	7	1.53
GSE84712	PCA	ATP-dependent chromatin remodeling	2.07E-04	6	1.31
GSE84712	PCA	neurotransmitter secretion	2.39E-04	8	1.74
GSE84712	PCA	positive regulation of mRNA splicing, via spliceosome	4.01E-04	5	1.09
GSE84712	PCA	gene silencing by RNA	4.19E-04	11	2.4
GSE84712	PCA	glutamate secretion	5.47E-04	6	1.31

GO TERM COLOUR LEGEND

- development
- differentiation
- proliferation
- cell cycle
- mitosis
- mitotic
- G1/S
- G2/M
- circadian
- genesis

Dataset/subtype conditioned highlighting

Dataset/subtype conditions highlighted

Neuron related

%Overlap Legend

20

10

0

Dataset	Method	GO term	P-Value	#Genes	%Overlap
GSE87375	dynDLT	cellular calcium ion homeostasis	3.48E-05	10	2
GSE87375	dynDLT	transport	6.68E-05	52	10.4
GSE87375	dynDLT	wound healing, spreading of epidermal cells	8.23E-04	4	0.8
GSE87375	ICA	antigen processing and presentation of exogenous peptide antigen via MHC class I, TAP-dependent	3.07E-07	9	1.8
GSE87375	ICA	proteolysis involved in cellular protein catabolic process	3.47E-04	8	1.6
GSE87375	ICA	neurotransmitter secretion	4.27E-04	6	1.2
GSE87375	ICA	cellular response to hormone stimulus	7.45E-04	7	1.4
GSE87375	NMF	vesicle-mediated transport	9.57E-12	28	5.6
GSE87375	NMF	transport	9.74E-11	94	18.8
GSE87375	NMF	tricarboxylic acid cycle	1.58E-09	11	2.2
GSE87375	NMF	protein folding	5.41E-09	19	3.8
GSE87375	NMF	ER to Golgi vesicle-mediated transport	1.12E-08	15	3
GSE87375	NMF	proteolysis involved in cellular protein catabolic process	1.93E-08	13	2.6
GSE87375	NMF	oxidation-reduction process	2.01E-08	45	9
GSE87375	NMF	intracellular protein transport	3.98E-08	24	4.8
GSE87375	NMF	antigen processing and presentation of exogenous peptide antigen via MHC class I, TAP-dependent	4.26E-08	10	2
GSE87375	NMF	cell-cell adhesion	4.89E-07	20	4
GSE87375	NMF	response to drug	2.88E-06	26	5.2
GSE87375	NMF	translation	6.38E-06	28	5.6
GSE87375	NMF	positive regulation of telomere maintenance via telomerase	1.43E-05	8	1.6
GSE87375	NMF	positive regulation of translation	1.44E-05	10	2
GSE87375	NMF	proton transport	1.67E-05	10	2
GSE87375	NMF	toxin transport	2.67E-05	8	1.6
GSE87375	NMF	positive regulation of protein localization to Cajal body	2.86E-05	5	1
GSE87375	NMF	protein transport	3.18E-05	34	6.8
GSE87375	NMF	cell redox homeostasis	4.26E-05	10	2
GSE87375	NMF	ubiquitin-dependent protein catabolic process	4.83E-05	15	3
GSE87375	NMF	protein stabilization	1.23E-04	13	2.6
GSE87375	NMF	negative regulation of apoptotic process	1.78E-04	31	6.2
GSE87375	NMF	NADH metabolic process	1.86E-04	5	1
GSE87375	NMF	RNA splicing	1.90E-04	18	3.6
GSE87375	NMF	cellular process	3.22E-04	6	1.2
GSE87375	NMF	positive regulation of telomerase RNA localization to Cajal body	4.82E-04	5	1
GSE87375	NMF	ATP metabolic process	5.29E-04	7	1.4
GSE87375	NMF	retrograde protein transport, ER to cytosol	6.30E-04	5	1
GSE87375	NMF	ER-associated ubiquitin-dependent protein catabolic process	6.43E-04	8	1.6
GSE87375	NMF	response to endoplasmic reticulum stress	7.66E-04	9	1.8
GSE87375	NMF	mRNA processing	7.73E-04	20	4
GSE87375	NMF	retrograde vesicle-mediated transport, Golgi to ER	8.08E-04	6	1.2
GSE87375	NMF	regulation of stress-activated MAPK cascade	8.76E-04	4	0.8
GSE87375	NMF	regulation of neuron projection development	9.49E-04	6	1.2
GSE87375	PCA	cell division	1.01E-35	65	13
GSE87375	PCA	mitotic nuclear division	3.90E-31	53	10.6
GSE87375	PCA	cell cycle	4.72E-30	74	14.8
GSE87375	PCA	chromosome segregation	2.54E-15	22	4.4
GSE87375	PCA	mitotic sister chromatid segregation	1.73E-09	10	2
GSE87375	PCA	mitotic chromosome condensation	3.06E-07	7	1.4
GSE87375	PCA	microtubule-based movement	2.93E-06	12	2.4
GSE87375	PCA	mitotic cytokinesis	6.22E-06	8	1.6
GSE87375	PCA	antigen processing and presentation of exogenous peptide antigen via MHC class I, TAP-dependent	6.22E-06	8	1.6
GSE87375	PCA	mitotic metaphase plate congression	1.51E-05	8	1.6
GSE87375	PCA	chromosome condensation	2.98E-05	6	1.2
GSE87375	PCA	attachment of spindle microtubules to kinetochore	4.03E-05	5	1
GSE87375	PCA	mitotic spindle organization	4.92E-05	7	1.4
GSE87375	PCA	protein localization to kinetochore	6.58E-05	5	1
GSE87375	PCA	proteolysis involved in cellular protein catabolic process	6.92E-05	9	1.8
GSE87375	PCA	metaphase plate congression	1.49E-04	5	1
GSE87375	PCA	cytokinesis	2.52E-04	7	1.4
GSE87375	PCA	mitotic spindle midzone assembly	2.75E-04	4	0.8
GSE87375	PCA	microtubule depolymerization	2.90E-04	5	1
GSE87375	PCA	regulation of attachment of spindle microtubules to kinetochore	4.72E-04	4	0.8
GSE87375	PCA	spindle organization	5.08E-04	5	1
GSE87375	PCA	mitotic spindle assembly checkpoint	8.21E-04	5	1

GO TERM COLOUR LEGEND

- development
- differentation
- proliferation
- cell cycle
- mitosis
- mitotic
- G1/S
- G2/M
- circadian
- genesis

Dataset/subtype conditioned highlighting

Dataset/subtype conditions highlighted

Pancreas related

%Overlap Legend

20

10

0

Dataset	Method	GO term	P-Value	#Genes	%Overlap
GSE92652	dynDLT	G-protein coupled receptor signaling pathway	1.63E-07	14	16.87
GSE92652	dynDLT	detection of chemical stimulus involved in sensory perception of smell	8.32E-07	10	12.05
GSE92652	ICA	G2/M transition of mitotic cell cycle	7.68E-07	16	3.64
GSE92652	ICA	cell division	2.79E-05	23	5.24
GSE92652	ICA	mitotic cytokinesis	4.76E-05	7	1.59
GSE92652	ICA	mitotic sister chromatid segregation	2.47E-04	6	1.37
GSE92652	ICA	inflammatory response	2.48E-04	22	5.01
GSE92652	ICA	mitotic nuclear division	2.53E-04	17	3.87
GSE92652	ICA	leukocyte migration	5.91E-04	11	2.51
GSE92652	ICA	mitotic spindle midzone assembly	6.53E-04	4	0.91
GSE92652	ICA	extracellular matrix organization	7.32E-04	14	3.19
GSE92652	NMF	viral transcription	1.71E-15	24	5.58
GSE92652	NMF	SRP-dependent cotranslational protein targeting to membrane	7.47E-12	19	4.42
GSE92652	NMF	translational initiation	9.12E-11	21	4.88
GSE92652	NMF	cell-cell adhesion	1.86E-09	27	6.28
GSE92652	NMF	nuclear-transcribed mRNA catabolic process, nonsense-mediated decay	3.36E-09	18	4.19
GSE92652	NMF	viral process	3.40E-09	28	6.51
GSE92652	NMF	mitotic nuclear envelope disassembly	7.81E-07	10	2.33
GSE92652	NMF	translation	3.07E-06	21	4.88
GSE92652	NMF	cell division	3.96E-06	25	5.81
GSE92652	NMF	rRNA processing	4.03E-06	19	4.42
GSE92652	NMF	sister chromatid cohesion	6.29E-06	13	3.02
GSE92652	NMF	IRE1-mediated unfolded protein response	1.01E-05	10	2.33
GSE92652	NMF	actin cytoskeleton organization	1.41E-05	14	3.26
GSE92652	NMF	intracellular transport of virus	2.56E-05	9	2.09
GSE92652	NMF	tRNA export from nucleus	9.56E-05	7	1.63
GSE92652	NMF	protein sumoylation	1.13E-04	12	2.79
GSE92652	NMF	regulation of glucose transport	1.15E-04	7	1.63
GSE92652	NMF	phagocytosis	1.33E-04	8	1.86
GSE92652	NMF	platelet degranulation	1.76E-04	11	2.56
GSE92652	NMF	ATP-dependent chromatin remodeling	1.79E-04	6	1.4
GSE92652	NMF	positive regulation of erythrocyte differentiation	2.21E-04	6	1.4
GSE92652	NMF	positive regulation of B cell differentiation	2.65E-04	5	1.16
GSE92652	NMF	mRNA processing	3.76E-04	14	3.26
GSE92652	NMF	response to endoplasmic reticulum stress	4.12E-04	9	2.09
GSE92652	NMF	G2/M transition of mitotic cell cycle	4.51E-04	12	2.79
GSE92652	NMF	mRNA splicing, via spliceosome	9.36E-04	15	3.49
GSE92652	PCA	cell division	3.91E-07	27	6.05
GSE92652	PCA	mitotic nuclear division	1.11E-04	18	4.04
GSE92652	PCA	G2/M transition of mitotic cell cycle	1.19E-04	13	2.91
GSE92652	PCA	mitotic metaphase plate congression	2.31E-04	7	1.57
GSE92652	PCA	microtubule bundle formation	2.81E-04	6	1.35
GSE92652	PCA	mitotic sister chromatid segregation	2.81E-04	6	1.35
GSE92652	PCA	response to oxidative stress	3.21E-04	11	2.47
GSE92652	PCA	interferon-gamma secretion	4.50E-04	4	0.9
GSE92652	PCA	protein phosphorylation	7.08E-04	24	5.38
GSE92652	PCA	extracellular matrix organization	9.48E-04	14	3.14

GO TERM COLOUR LEGEND

development
differentation
proliferation
cell cycle
mitosis
mitotic
G1/S
G2/M
circadian
genesis

Dataset/subtype conditioned highlitghing

Dataset/subtype conditions highlighted

Blood cell related

%Overlap Legend

20
10
0

Dataset	Method	GO term	P-Value	#Genes	%Overlap
EMTAB2565	dynDLT	suberin biosynthetic process	5.74E-08	8	1.6
EMTAB2565	dynDLT	oxidation-reduction process	3.20E-04	47	9.4
EMTAB2565	dynDLT	iron ion homeostasis	4.43E-04	6	1.2
EMTAB2565	dynDLT	cellular response to nitric oxide	6.41E-04	5	1
EMTAB2565	ICA	translation	1.07E-20	79	15.8
EMTAB2565	ICA	ribosome biogenesis	4.66E-15	25	5
EMTAB2565	ICA	cytoplasmic translation	8.15E-10	13	2.6
EMTAB2565	ICA	ribosomal small subunit assembly	3.88E-04	7	1.4
EMTAB2565	ICA	protein transport	4.35E-04	19	3.8
EMTAB2565	NMF	response to cadmium ion	7.67E-26	51	10.2
EMTAB2565	NMF	oxidation-reduction process	3.25E-23	95	19
EMTAB2565	NMF	response to oxidative stress	1.58E-22	44	8.8
EMTAB2565	NMF	response to salt stress	5.93E-17	48	9.6
EMTAB2565	NMF	hydrogen peroxide catabolic process	1.70E-12	19	3.8
EMTAB2565	NMF	response to cytokinin	6.59E-12	25	5
EMTAB2565	NMF	response to karrikin	9.30E-10	19	3.8
EMTAB2565	NMF	response to cold	1.80E-09	28	5.6
EMTAB2565	NMF	lignin biosynthetic process	4.49E-09	14	2.8
EMTAB2565	NMF	ATP hydrolysis coupled proton transport	1.38E-08	11	2.2
EMTAB2565	NMF	response to water deprivation	3.72E-08	25	5
EMTAB2565	NMF	plant-type cell wall organization	1.22E-06	13	2.6
EMTAB2565	NMF	proton transport	1.52E-06	8	1.6
EMTAB2565	NMF	tricarboxylic acid cycle	3.14E-06	10	2
EMTAB2565	NMF	response to wounding	4.04E-06	18	3.6
EMTAB2565	NMF	electron transport chain	5.98E-06	7	1.4
EMTAB2565	NMF	toxin catabolic process	9.28E-06	9	1.8
EMTAB2565	NMF	glutathione metabolic process	1.20E-05	10	2
EMTAB2565	NMF	phenylpropanoid biosynthetic process	1.30E-05	8	1.6
EMTAB2565	NMF	response to abscisic acid	1.73E-05	25	5
EMTAB2565	NMF	cellular water homeostasis	1.91E-05	8	1.6
EMTAB2565	NMF	ion transmembrane transport	3.62E-05	7	1.4
EMTAB2565	NMF	glycolytic process	3.66E-05	10	2
EMTAB2565	NMF	water transport	8.20E-05	5	1
EMTAB2565	NMF	mitochondrial electron transport, ubiquinol to cytochrome c	1.21E-04	5	1
EMTAB2565	NMF	response to water	1.21E-04	5	1
EMTAB2565	NMF	cold acclimation	1.62E-04	8	1.6
EMTAB2565	NMF	response to zinc ion	2.08E-04	8	1.6
EMTAB2565	NMF	ATP synthesis coupled proton transport	2.53E-04	7	1.4
EMTAB2565	NMF	aging	3.35E-04	7	1.4
EMTAB2565	NMF	response to UV-B	5.02E-04	8	1.6
EMTAB2565	NMF	proteolysis involved in cellular protein catabolic process	6.43E-04	10	2
EMTAB2565	NMF	defense response to bacterium	7.39E-04	17	3.4
EMTAB2565	PCA	translation	4.43E-25	86	17.2
EMTAB2565	PCA	ribosome biogenesis	2.38E-20	30	6
EMTAB2565	PCA	cytoplasmic translation	5.34E-11	14	2.8
EMTAB2565	PCA	ribosomal small subunit assembly	4.61E-05	8	1.6
EMTAB2565	PCA	ribosomal large subunit assembly	8.67E-04	6	1.2

GO TERM COLOUR LEGEND

development
differentation
proliferation
cell cycle
mitosis
mitotic
G1/S
G2/M
circadian
genesis

Dataset/subtype conditioned highlighting

Dataset/subtype conditions highlighted

Plant related

%Overlap Legend

20
10
0

Dataset	Method	GO term	P-Value	#Genes	%Overlap
EMTAB6811	dynDLT	sodium ion transport	2.50E-10	16	3.2
EMTAB6811	dynDLT	transmembrane transport	2.98E-08	21	4.2
EMTAB6811	dynDLT	excretion	3.07E-07	7	1.4
EMTAB6811	dynDLT	kidney development	2.64E-06	15	3
EMTAB6811	dynDLT	sodium-independent organic anion transport	4.06E-06	7	1.4
EMTAB6811	dynDLT	receptor-mediated endocytosis	5.33E-06	12	2.4
EMTAB6811	dynDLT	regulation of pH	5.40E-06	7	1.4
EMTAB6811	dynDLT	sodium ion transmembrane transport	9.09E-05	9	1.8
EMTAB6811	dynDLT	regulation of microvillus length	1.64E-04	4	0.8
EMTAB6811	dynDLT	inorganic anion transport	2.62E-04	5	1
EMTAB6811	dynDLT	ion transport	2.62E-04	7	1.4
EMTAB6811	dynDLT	proteolysis	3.24E-04	23	4.6
EMTAB6811	dynDLT	cellular response to hepatocyte growth factor stimulus	3.36E-04	5	1
EMTAB6811	dynDLT	multicellular organismal water homeostasis	6.58E-04	4	0.8
EMTAB6811	dynDLT	glutathione biosynthetic process	9.25E-04	4	0.8
EMTAB6811	ICA	spermatid development	6.93E-05	10	2
EMTAB6811	ICA	spermatogenesis	5.04E-04	17	3.4
EMTAB6811	ICA	sperm motility	7.55E-04	7	1.4
EMTAB6811	ICA	regulation of nucleic acid-templated transcription	8.12E-04	5	1
EMTAB6811	ICA	negative regulation of mRNA splicing, via spliceosome	9.40E-04	5	1
EMTAB6811	NMF	translation	8.65E-22	52	10.4
EMTAB6811	NMF	positive regulation of protein localization to Cajal body	7.55E-11	8	1.6
EMTAB6811	NMF	cell-cell adhesion	9.87E-11	27	5.4
EMTAB6811	NMF	mRNA processing	2.10E-10	21	4.2
EMTAB6811	NMF	RNA splicing	3.27E-10	18	3.6
EMTAB6811	NMF	positive regulation of establishment of protein localization to telomere	2.85E-08	7	1.4
EMTAB6811	NMF	positive regulation of telomerase RNA localization to Cajal body	5.15E-08	8	1.6
EMTAB6811	NMF	cell division	7.74E-08	21	4.2
EMTAB6811	NMF	translational initiation	1.04E-07	12	2.4
EMTAB6811	NMF	transcription, DNA-templated	1.06E-07	49	9.8
EMTAB6811	NMF	formation of translation preinitiation complex	1.29E-07	9	1.8
EMTAB6811	NMF	ATP-dependent chromatin remodeling	1.29E-07	9	1.8
EMTAB6811	NMF	negative regulation of mRNA splicing, via spliceosome	3.62E-07	9	1.8
EMTAB6811	NMF	chromatin remodeling	5.94E-07	13	2.6
EMTAB6811	NMF	IRES-dependent viral translational initiation	7.17E-07	6	1.2
EMTAB6811	NMF	toxin transport	8.79E-07	10	2
EMTAB6811	NMF	negative regulation of transcription, DNA-templated	1.08E-06	36	7.2
EMTAB6811	NMF	regulation of translational initiation	1.17E-06	9	1.8
EMTAB6811	NMF	covalent chromatin modification	1.47E-06	11	2.2
EMTAB6811	NMF	liver regeneration	1.76E-06	11	2.2
EMTAB6811	NMF	mRNA splicing, via spliceosome	2.16E-06	14	2.8
EMTAB6811	NMF	protein stabilization	2.19E-06	16	3.2
EMTAB6811	NMF	binding of sperm to zona pellucida	3.99E-06	9	1.8
EMTAB6811	NMF	regulation of circadian rhythm	4.46E-06	10	2
EMTAB6811	NMF	positive regulation of translation	7.34E-06	11	2.2
EMTAB6811	NMF	protein folding	9.71E-06	14	2.8
EMTAB6811	NMF	positive regulation of telomere maintenance via telomerase	2.25E-05	8	1.6
EMTAB6811	NMF	DNA unwinding involved in DNA replication	3.30E-05	5	1
EMTAB6811	NMF	circadian regulation of gene expression	3.38E-05	10	2
EMTAB6811	NMF	neural tube closure	4.33E-05	12	2.4
EMTAB6811	NMF	nucleosome assembly	4.33E-05	12	2.4
EMTAB6811	NMF	DNA repair	4.91E-05	16	3.2
EMTAB6811	NMF	cellular response to DNA damage stimulus	5.51E-05	19	3.8
EMTAB6811	NMF	cell proliferation	5.83E-05	19	3.8
EMTAB6811	NMF	cell migration	8.63E-05	17	3.4
EMTAB6811	NMF	cell cycle	9.37E-05	13	2.6
EMTAB6811	NMF	regulation of translation	1.17E-04	9	1.8
EMTAB6811	NMF	negative regulation of transcription from RNA polymerase II promoter	1.24E-04	39	7.8
EMTAB6811	NMF	regulation of alternative mRNA splicing, via spliceosome	1.35E-04	8	1.6
EMTAB6811	NMF	brain development	2.15E-04	21	4.2
EMTAB6811	NMF	cellular response to transforming growth factor beta stimulus	2.62E-04	10	2
EMTAB6811	NMF	cellular response to X-ray	3.03E-04	5	1
EMTAB6811	NMF	nucleosome disassembly	3.03E-04	5	1
EMTAB6811	NMF	cytoplasmic translation	3.37E-04	9	1.8
EMTAB6811	NMF	cerebral cortex development	3.48E-04	10	2
EMTAB6811	NMF	osteoblast differentiation	3.63E-04	12	2.4
EMTAB6811	NMF	negative regulation of catalytic activity	4.60E-04	9	1.8
EMTAB6811	NMF	response to drug	5.16E-04	29	5.8
EMTAB6811	NMF	rRNA processing	5.60E-04	9	1.8
EMTAB6811	NMF	spindle organization	7.23E-04	5	1
EMTAB6811	NMF	nucleocytoplasmic transport	8.07E-04	6	1.2
EMTAB6811	NMF	protein import into nucleus	8.88E-04	8	1.6
EMTAB6811	NMF	regulation of cell migration	9.73E-04	9	1.8
EMTAB6811	PCA	spermatogenesis	6.48E-05	19	3.8
EMTAB6811	PCA	spermatid development	4.41E-04	9	1.8
EMTAB6811	PCA	tRNA wobble uridine modification	5.13E-04	4	0.8

GO TERM COLOUR LEGEND

development
differentation
proliferation
cell cycle
mitosis
mitotic
G1/S
G2/M
circadian
genesis

Dataset/subtype conditioned highlighting

Dataset/subtype conditions highlighted

Stem cell/ tissue type related

%Overlap Legend

20
10
0
